# Interaction of a Short Peptide with G-Quadruplex-Forming Sequences: An SRCD and CD Study

**DOI:** 10.3390/pharmaceutics13081104

**Published:** 2021-07-21

**Authors:** Claudia Honisch, Eugenio Ragazzi, Rohanah Hussain, John Brazier, Giuliano Siligardi, Paolo Ruzza

**Affiliations:** 1 Institute of Biomolecular Chemistry of CNR, Via F. Marzolo, 1, 35131 Padova, Italy; claudiahonisch@gmail.com; 2 Department of Chemical Sciences, University of Padua, Via F. Marzolo, 1, 35131 Padova, Italy; 3 Department of Pharmaceutical and Pharmacological Sciences, University of Padua, Largo Meneghetti, 2, 35131 Padova, Italy; eugenio.ragazzi@unipd.it; 4 Diamond Light Source Ltd., Harwell Science and Innovation Campus, Didcot OX11 0DE, UK; rohanah.hussain@diamond.ac.uk (R.H.); giuliano.siligardi@diamond.ac.uk (G.S.); 5 School of Pharmacy, University of Reading, Reading RG6 6DX, UK; j.a.brazier@reading.ac.uk

**Keywords:** G-quadruplex DNA, peptide, photo-stability

## Abstract

G-quadruplex (G4) forming DNA sequences were recently found to play a crucial role in the regulation of genomic processes such as replication, transcription and translation, also related to serious diseases. Therefore, systems capable of controlling DNA and RNA G-quadruplex structures would be useful for the modulation of various cellular events. In particular, peptides represent good candidates for targeting G-quadruplex structures, since they are easily tailored to enhance their functionality. In this work, we analyzed, by circular dichroism and synchrotron radiation circular dichroism spectroscopies, the interaction of a 25-residue peptide deriving from RHAU helicases (***Rhau25***) with three G-quadruplex-forming oligonucleotide sequences, in both sodium- and potassium-containing buffers, the most relevant monovalent cations in physiological conditions. The peptide displayed greater affinity for the G4 sequences adopting a parallel structure. However, it showed the ability to also interact with antiparallel or hybrid G-quadruplex structures, inducing a conformation conversion to the parallel structure. The stability of the oligonucleotide structure alone or in presence of the ***Rhau25*** peptide was studied by temperature melting and UV denaturation experiments, and the data showed that the interaction with the peptide stabilized the conformation of oligonucleotide sequences when subjected to stress conditions.

## 1. Introduction

**Figure 1:**
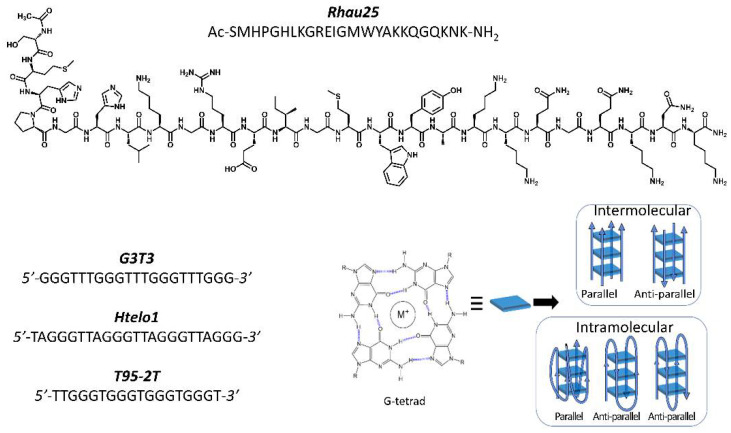
Sequences of the synthetized ***Rhau25*** peptide and *G3T3*, *Htelo1* and *T95-2T* G-quadruplex-forming oligonucleotide sequences with cartoons of the peptide ***Rhau25***, G-tetrad and G4 topologies.

G-quadruplex (G4) nucleic acid structures, present in guanine-rich nucleic acid sequences, result from the propensity of these sequences to form atypical and thermodynamically stable structures under physiological conditions formed by stacks of Hoogsteen-bonded guanine tetrads (Figure 1) [1]. These highly conserved structures, found in both DNA and RNA nucleic acids, have a regulatory role in replication, transcription and recombination [2]. Moreover, G-quadruplex-forming sequences have been also found in viruses [3], bacteria [4] and protozoa [5]. Indeed, studies have shown that G-quadruplex structures play a role in the control of the human immunodeficiency virus-1 [3,6,7,8,9], of the Epstein-Barr virus [10], of the human papilloma virus (HPVs) [11], and of Zika virus [12]. Recently, it has been found that G-quadruplex structures are also present in the novel SARS-CoV-2 coronavirus [13].

The highest abundance of putative G-quadruplexes sequences is located at telomeres [14], which protect chromosomes from degradation, end-to-end fusions, and are recognized as double-strand break sites [15]. In most telomeric DNAs, guanines and cytosines are distributed asymmetrically between the two DNA strands, with the G-rich strand running 5′ to 3′ from the centromere to the telomere [16].

Systems capable of controlling DNA and RNA G-quadruplex structures would be useful for the modulation of various cellular events and different G-quadruplex-targeting ligands have been described [17,18], including phthalocyanine [19], porphyrin [20], and other derivatives [21,22]. From these, peptides represent a class of highly specific ligands with a greater degree of functionality including binding on-off switching, cellular penetration, and the ability to target organelles [23,24,25,26,27]. Among them, the N-terminal domain of the RNA helicase associated with AU-rich element (RHAU), a member of the human DEAH (Asp-Glu-Ala-His) box family of RNA helicases, which includes a specific motif, named RSM, necessary for G-quadruplex recognition and interaction (aa 54–66) [28], has recently raised the interest of several authors [29,30,31,32].

With the aim to develop a peptide that selectively interacts with secondary G4 structures we synthesized a peptide, named ***Rhau25*** (Figure 1), containing the sequence 52–75 of the RHAU protein. The binding of this peptide to G-quadruplex-forming sequences as well as the structure of oligonucleotide sequences and the stability of peptide/oligonucleotide complexes have been evaluated by circular dichroism (CD) spectroscopy using benchtop CD instruments and Diamond B23 beamline for synchrotron radiation circular dichroism (SRCD). This chiroptical spectroscopy is a useful tool for the characterization of G-quadruplex structures and nucleic acids-peptides interactions. Spectroscopic studies were performed in the presence of either sodium or potassium ions, the physiologically relevant monovalent ions involved in the stabilization of cationic coordination with the oxygen atom of the carbonyl group (O6) of guanine.

Metal ion-G4 interaction studies indicated that the position of these ions could be within the quartet plane or between the planes of the quartet depending on ionic radius. Potassium ion (ionic radius 1.33 Å) is too large to be coordinated in the plane of G-quartet, whereas Na^+^ (ionic radius 0.95 Å) is small to be coordinated within the plane of G-quartet. Moreover, these studies indicated that K^+^ stabilizes more efficiently G4 than sodium ion, and that G4 structures exhibit diverse topologies depending on the monovalent cation added [33].

Three G-quadruplex-forming sequences (Figure 1), including the human telomeric sequence *Htelo1*, the *T95-2T* sequence, and a synthetic sequence named *G3T3*, have been selected to carry out interaction studies.

In this work, we showed that the selected peptide displays greater affinity for G-quadruplex sequences adopting a parallel conformation, stabilizing this structure towards stress conditions as heating and UV radiation. Moreover, the interaction of the peptide with the oligonucleotide sequence adopting antiparallel or hybrid structures induced a conformational conversion to a parallel G4-structure in the presence of both examined ions.

## 2. Materials and Methods

Oligonucleotide Preparation. Synthetic guanine-rich oligonucleotides were purchased from Eurogentec (Seraing, Belgium). The deoxyoligonucleotides were dissolved in water and allowed to equilibrate overnight at 4 °C prior to their use; stock solutions were filtered using 0.45 μm Millipore syringe filters, and the concentrations were determined by UV−visible spectrophotometry at 90 °C to ensure that any secondary structure was fully denatured. G-quadruplex forming sequences were then diluted to the desired concentration in 10 mM Tris-HCl buffer, pH 7.4, in presence of 70 mM potassium or sodium ions without annealing. Calf Thymus DNA (ctDNA) sodium salt was purchased from Sigma Aldrich (Milan, Italy) and used without further purification. ctDNA was dissolved in 10 mM Tris-HCl buffer, pH 7.4, and stirred overnight to allow complete dissolution. The purity of ctDNA was checked by measuring the ratio of A_260_/A_280_ = 1.82, and the concentration of ctDNA was determined by the absorption of ctDNA at 260 nm (ε_260_ = 6600 L mol^−1^ cm^−1^).

Peptide synthesis. The solid phase peptide synthesis (SPPS) was achieved with the Fmoc/HBTU chemistry approach [34] and carried out automatically using a Biotage^®^ Syro Wave™ synthesizer (Biotage AB, Uppsala, Sweeden) controlled by Syro XP peptide software. After acetylation by treatment with acetic anhydride, the peptide was detached from the Rink-amide resin along with removal of the side-chain protecting groups by treatment with TFA in presence of TIS and water as scavengers. The peptide was isolated by addition of ethyl ether and purified by elution on a Dionex Vydac reverse phase C18 300 Å, 10 µ, 22 × 250 mm column using a preparative Shimadzu HPLC system (Kyoto, Japan) equipped with LC-8A pumps, SLC-8A controller, an SPD-6A spectrophotometric detector, and an ERC-3562 ERMA degasser. LC-ESI-MS analyses were conducted using an Agilent 1260 Infinity II analytical HPLC system (G7129A vialsampler, G7117C DAD HS, and G7111B Quat. Pump) equipped with a 6130 Quadrupole LC-MS analyzer. The calculated mass was 2951.41 Da.

Circular Dichroism. CD spectra were acquired on a Jasco J-1500 CD spectrometer equipped with a Jasco PTC-423S temperature controller (Jasco International, Tokyo, Japan). Far-UV CD spectra were collected in 0.1 cm pathlength quartz cuvettes (Hellma Analytics, Southend on See, UK) at 25 °C in the 198–360 nm range, at 100 nm/min scanning speed, 1 s response time, 2 nm bandwidth, 0.5 nm data pitch. The spectra recorded were the average of 4 scans. Peptide concentration was 0.09 mg/mL in the 10 mM Tris-HCl buffer; pH 7.4. G4 titration was conducted, adding aliquots of 2 µL ***Rhau25*** peptide 0.29 mM stock solution up to 2.2 molar equivalents to 200 µL of 13.5 µM oligonucleotide. Spectra were accordingly corrected for dilution.

Synchrotron Radiation Circular Dichroism. SRCD melting experiments were performed in the 10–90 °C range with 5 °C steps and allowing 2 min equilibration time. Melting and UV-denaturation measurements were collected on Module A of beamline B23 of Diamond Light Source Ltd synchrotron facility, Harwell Science and Innovation Campus (Didcot, UK). Spectra were recorded in the 198–360 nm range in 0.1 cm pathlength quartz cuvettes (Hellma Analytics). Peptide concentration was 0.09 mg/mL in 10 mM Tris-HCl buffer, pH 7.4. 

CD and SRCD spectra were plotted and analyzed using OriginPro2018 software (OriginLab Corporation, Northampton, MA, USA). The *K_d_* values of peptide/G-quadruplex complexes were determined by fitting the titration curves with the Hill equation (Hill1 function in OriginPro2018 software). Multivariate analysis of spectra was obtained with JMP Pro software version 15 (SAS Institute Inc., Cary, NC, USA).

## 3. Results and Discussion

### 3.1. Peptide Design and Secondary Structure

The ***Rhau25*** peptide was synthesized by solid phase peptide synthesis (SPPS) using Fmoc/HBTU chemistry, and then purified by preparative RP-HPLC and characterized by LC-ESI-MS (Figure S1 in Supplementary Materials).

**Figure 2:**
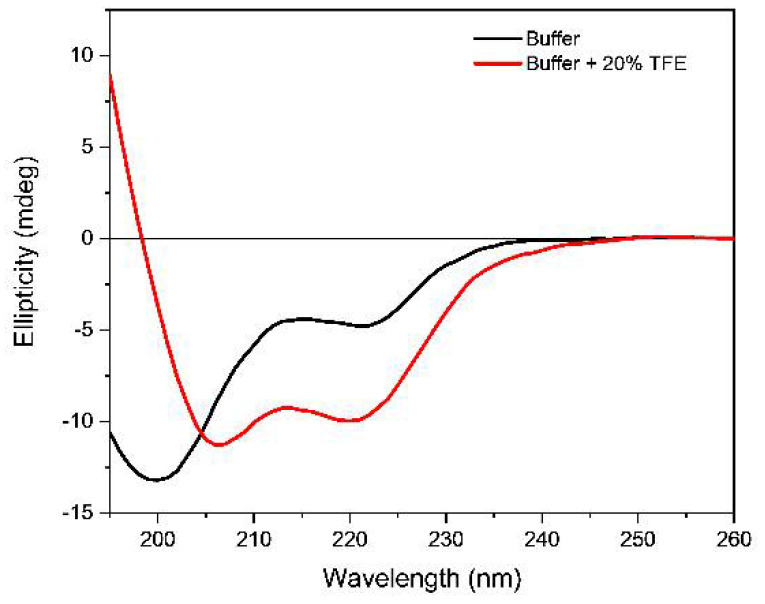
Far-UV CD spectra of ***Rhau25*** peptide (0.09 mg/mL or 30.5 μM) in 10 mM Tris-HCl buffer, pH 7.4, (black line) and in presence of 20% *v*/*v* trifluoroethanol (red line).

The secondary structure of the peptide was evaluated by CD spectroscopy in 10 mM Tris-HCl buffer, pH 7.4, in presence of 70 mM either sodium or potassium ions. In these conditions, the far-UV CD spectrum of peptide is characterized by the presence of two negative bands at about 200 and 222 nm (Figure 2), suggesting the presence of an ensemble of a secondary structure as confirmed by the estimation of the secondary structure content (SSE) by the Jasco software. The addition of trifluoroethanol (20%) induced a change in the shape of the CD spectrum (Figure 2) and the resulting spectrum adopted the typical profile of α-helix as confirmed by the SSE analysis in agreement with the solution structure determined by NMR method of an analogue peptide corresponding to sequences 1–20 of our peptide [28]. The NMR study showed that the peptide adopted an L-shaped structure, containing an α-helix spanning from Gly5 to Ala17, and that this structure was retained when the peptide bound to G-quadruplex sequences [28].

In the data obtained by CD spectroscopy the peptide conformational signature overlaps with those of the G-quadruplex bases and the puckering of the deoxyribose components of the nucleosides in the far-UV spectral region (185–250 nm). In the near-UV region (250–320 nm), on the other hand, due to the negligible absorption of the Trp and Tyr residues (about 0.021 with peptide concentration of 30 µM), only the conformation of the stacked nucleotide bases are detected that can be successfully used to identify the G-quadruplex topologies.

**Figure 3:**
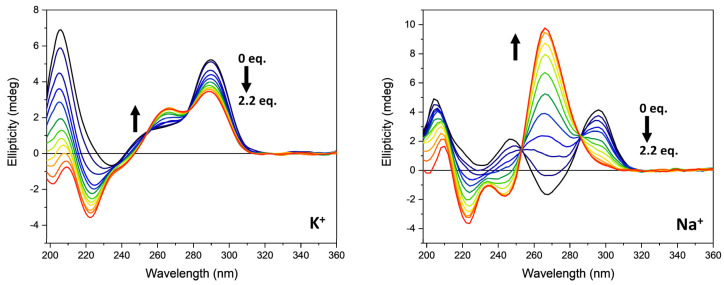
CD spectra of *G3T3* (13.5 µM) in the absence (black line) and in presence of increasing amounts of ***Rhau25*** peptide in 10 mM Tris-HCl buffer, pH 7.4, 70 mM potassium (**left**) or sodium ions (**right**). The arrows represent the direction of the spectral change as the peptide concentration is increased.

**Figure 4:**
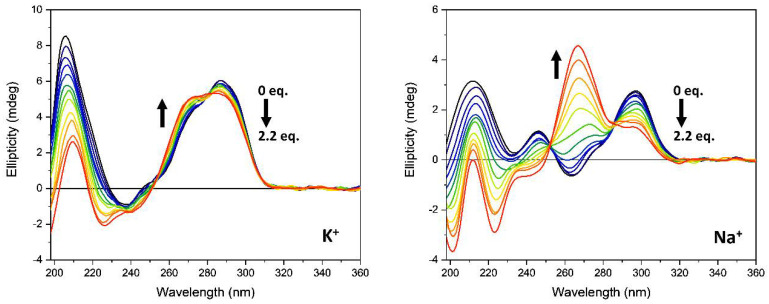
CD spectra of *Htelo1* G-quadruplex (13.5 µM) in the absence (black line) and in presence of increasing amounts of ***Rhau25*** peptide in 10 mM Tris-HCl buffer, pH 7.4, 70 mM potassium (**left**) or sodium (**right**) ions. The arrows represent the direction of the signal change as the peptide concentration is increased.

**Figure 5:**
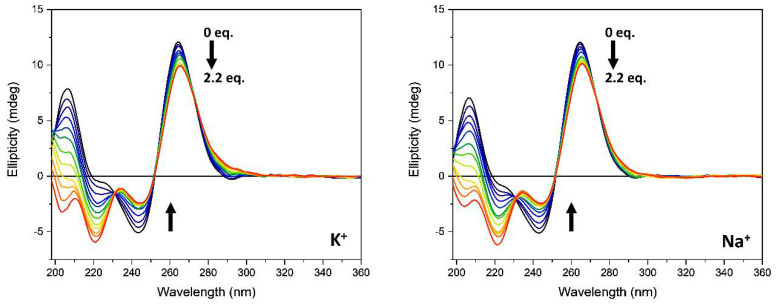
CD spectra of *T95-2T* G-quadruplex (13.5 µM) in the absence (black line) and in the presence of increased amounts of ***Rhau25*** peptide in 10 mM Tris-HCl buffer, pH 7.4, 70 mM potassium (**left**) or sodium (**right**) ions. The arrows represent the direction of the spectral change as the peptide concentration is increased.

The determination of the bound conformation of the peptide when bound to the G-quadruplex can only be conducted, at best, qualitatively, as the conformation of the G-quadruplex molecules is induced to different degrees by the binding of the ***Rhau25*** peptide as demonstrated in Figure 3, Figure 4 and Figure 5. To do this, the CD spectrum of the complex between the G4 and the peptide at (1:1.8) molar ratio was subtracted from that of the complex with the highest peptide molar ratio of (1:2.2) as illustrated in Figure S4. This is a qualitative assessment as any residual spectral feature in the near-UV region will indicate that the base contributions have not been fully cancelled out, hence making this analysis less accurate. Nevertheless, the results appear to indicate that the conformation of the bound peptide to the G4s for 5 of the 6 complexes are of the more unordered conformation observed for the ***Rhau25*** peptide in Tris-HCl buffer, while the peptide bound to *Htelo1* G-quadruplex sequence appears to bind with a more α-helical conformation, though the base contributions are quite significant. This is better illustrated by comparing the ratios of the CD intensity at 202 nm over that at 220 nm for the calculated spectra of Figure S4. 

### 3.2. Peptide G-Quadruplex Interaction

The ability of the ***Rhau25*** peptide to bind to *G3T3*, *Htelo1*, and *T95-2T* G-quadruplex-forming sequences was assessed by CD spectroscopy in the presence of either sodium or potassium ions.

CD spectroscopy, besides allowing for the evaluation of the peptide-G4 interaction, provides useful information on the structure of the oligonucleotide sequence itself, being the 240–300 nm region diagnostic of the G-quadruplex topology [35,36,37,38]. Indeed, a parallel G4 structure is characterized by the presence of a positive band at about 264 nm and a negative band at 245 nm, while antiparallel structure shows a positive band at 295 nm and a negative band at 260 nm [35,37]. Hybrid or 3 + 1 structures show two positive bands at 295 and 260 nm, and a negative band at 245 nm [38].

As shown in Figure 3, the CD spectrum of *G3T3* in presence of 70 mM potassium ions is characterized by a positive band at 290 nm with a shoulder at about 254 nm, a negative band at 234 nm, and a strong positive band at 205 nm, suggesting the presence of a hybrid or 3 + 1 structure. The replacement of potassium with sodium ions induced a drastic change in the CD pattern of *G3T3* sequence that is characterized by two positive bands at 296 and 246 nm, respectively, and a negative band at 268 nm. The strong positive band at 205 nm observed in the presence of potassium ions is still present, but of lower intensity. This spectral feature is typically the CD profile of an antiparallel G4 structure.

The addition of the ***Rhau25*** peptide influenced the CD spectra of the *G3T3* sequence in the function of the monovalent cation added to the buffer. In the presence of potassium ions, a decrease in the intensity of the positive band at about 290 nm accompanied by the appearance of a positive band at about 265 nm that replaces the shoulder previously described characterized the CD spectrum when the peptide reached a 2.2 peptide/G4 molar ratio (Figure 3, left panel). At wavelengths lower than 240 nm the dichroic signal contains the contribution of both peptide and G4 sequences and is characterized by the presence of two negative bands at 221 and 200 nm (Figure 3, left panel). The addition of the ***Rhau25*** peptide in the presence of sodium ions drastically modified the CD spectrum of *G3T3*. At the same peptide-G4 molar ratio, an intense peak at 266 nm accompanied by a negative band at 244 nm appeared, suggesting a conformational conversion of *G3T3* sequence to a parallel quadruplex structure (Figure 3, right). Although the far-UV region (190–240 nm) also contains the CD contribution of the peptide, the G4 CD contribution is the dominating one, showing a negative band at about 222 nm with a positive band at 210 nm that is qualitatively similar to that observed in the presence of potassium ions (Figure 3, left). For the G4, the CD contribution in the far-UV region is due to the puckering of the sugar moiety as well as the nucleoside chromophore, whereas the in the near-UV region, it is solely due to the nucleoside electronic transitions. The CD contribution of the peptide, on the other hand, is mainly in the investigated far-UV region below 240 nm (Figure 2) and negligible in the near-UV region due to the aromatic side-chain of Tyr and Trp residues.

Similarly, the *Htelo1* structure was affected by the monovalent cation composition (Figure 4). According to literature data, in the presence of sodium ions, the human telomeric sequence adopts an antiparallel structure [39], while in the presence of potassium ions, the CD spectrum is characterized by a positive band at 287 nm with a shoulder at 274 and a negative band at 238 nm (Figure 4, left). The *Htelo1* structure in K^+^ solution, on the other hand, had not been as well identified as that in Na^+^ solution and was suggested to contain both antiparallel and parallel arrangements [40,41,42,43].

The addition of ***Rhau25*** peptide to the *Htelo1* sequence, in the presence of K^+^, produced minimal modification of the CD spectrum in the G4 diagnostic region (Figure 4, left), while a decreased intensity of the positive band at 206 nm was observed. On the other hand, in the presence of sodium ions (Figure 4, right), the peak at 290 nm disappeared in the presence of the ***Rhau25*** peptide and a conformational conversion to a parallel structure occurred, as revealed by the positive band at 265 nm which was qualitatively similar to that observed for the *G3T3* under the same conditions (Figure 3, right).

The CD spectrum of *T95-2T* was unaffected by the nature of the monovalent ions added to the buffer and was characterized by the presence of a strong positive band at about 266 nm and a negative band at about 245 nm. An additional positive band was observed at 206 nm in both the explored conditions (Figure 5). The addition of the ***Rhau25*** peptide modified the G-quadruplex *T95-2T* spectral features in the same manner in the presence of either sodium or potassium ions. In both titrations a decreased intensity of both positive and negative bands at 266 and 245 nm, respectively, were observed indicative of a preserved G-quadruplex parallel structure topology. In the far-UV region below 240 nm, two negative bands at 203 and 221 nm with similar spectral changes were observed for the titrations in both sodium and potassium ion solutions.

The apparent *K_d_* values of peptide/G-quadruplex complexes in either sodium or potassium ions were determined by fitting the titration curves with the Hill equation [44]. The calculated *K_d_* values of the investigated G-quadruplex sequences with the ***Rhau25*** peptide in both KCl and NaCl conditions, respectively, are summarized in Table 1. *Htelo* appears to bind to ***Rhau25*** peptide with less affinity, demonstrating about half that of *G3T3* and *T95*-*2T*, which showed similar binding affinities. The cooperativity for all titrations appears to be between 1.5 and 2, indicating that, in all six cases, the binding of the peptide increases the G-quadruplex affinity as more ligand was bound to it, while the stoichiometry appears to be 1:1.

In addition, to evaluate the selectivity of ***Rhau25*** peptide for G4 structures compared to double-stranded DNA molecules, the peptide was titrated with calf thymus DNA (ctDNA). No significant changes in the shape of the peptide spectrum were observed, indicating the lack of interactions with ctDNA, which was successively confirmed by melting and UV-denaturation studies on the ***Rhau25*** peptide alone or in presence of ctDNA (Figure S2 in Supplementary Materials).

### 3.3. Multivariate Statistical Analysis of G-Quadruplex Structure

Multivariate analysis of CD spectra can offer a satisfactory and reliable evaluation of G-quadruplex topologies such as parallel, antiparallel and so-called “hybrid” [35]. Principal component analysis (PCA) and hierarchical cluster analysis (HCA) were used to obtain an unbiased classification of G-quadruplex structures, independent of simple visual evaluation of the CD spectra with and without the ***Rhau25*** peptide.

To this purpose, a library of 23 CD spectra of various G4 sequences of known high-resolution structures from NMR and X-ray of deposited Protein Data Base (pdb) data files was used as the reference base data set and for the multivariate analysis [35].

**Figure 6:**
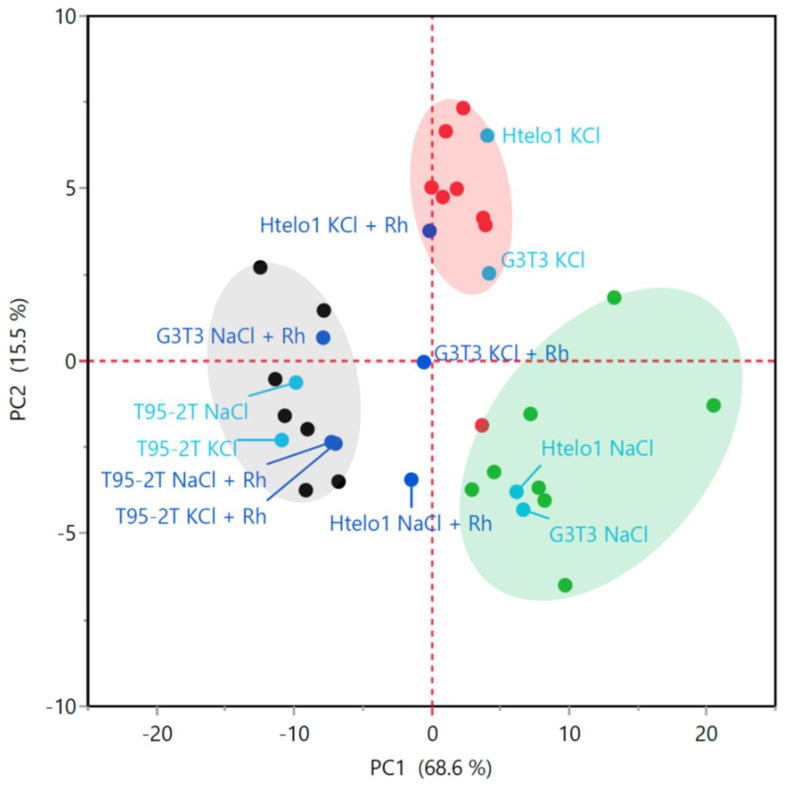
PCA plot of the first and second principal components. In parentheses is the fraction of total variance explained. The blue solid circles represent *G3T3*, *Htelo1*, and *T95-2T* with and without ***Rhau25*** (Rh) in Na^+^ and K^+^ respectively (total of 12 samples). The three clusters represent the parallel (grey), antiparallel (green) and hybrid (red) topologies defined by the reference pdb data (black solid circles for grey cluster, green solid circles for green cluster and red solid circles for red cluster [35]). The analysis was carried out using the most distinctive interval of wavelengths (220–310 nm) of the CD spectra.

Figure 6 shows the result of PCA conducted on the CD spectra of reference data together with the 12 experimental conditions herein investigated. Ten out of the 12 investigated data fall in the three main clusters observed in Villar-Guerra et al. [35] indicative of parallel, antiparallel and hybrid G4 topologies (Figure 6). However, *Htelo1* with ***Rhau25*** in Na^+^, and *G3T3* with ***Rhau25*** in K^+^ did not fall into any of the three main groups (Figure 6). 

**Figure 7:**
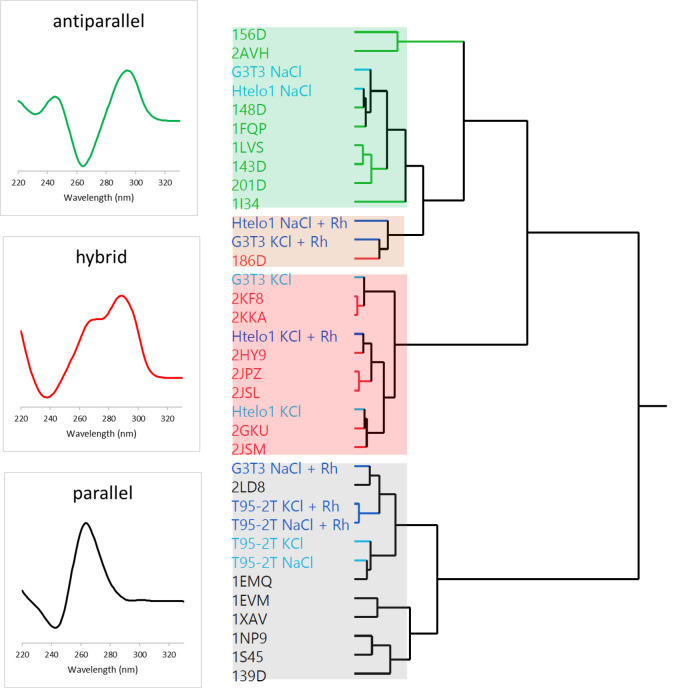
Hierarchical cluster analysis on the CD spectra of experimental samples, evaluated with reference spectra from [35]. The dendrogram on the right indicates three main clusters, comparable to those found with PCA analysis illustrated in Figure 6. For each cluster, a typical associated CD reference spectrum is shown, indicating antiparallel, “hybrid”, and parallel G4 topologies, respectively. A further cluster highlighted in amber color includes the spectra of intermediate characteristics. Cluster analysis was obtained according to Ward’s minimum variance method using the data of the most distinctive spectra interval of 250–300 nm.

Hierarchical cluster analysis confirmed the assignment of the 12 CD spectra to the G4 structure characteristics (Figure 7). For the samples studied here, besides the assignment to parallel and antiparallel classes, the cluster analysis was able to distinguish the “hybrid” class, a further additional group that included the spectra of *Htelo1* with ***Rhau25*** in Na^+^, and that of *G3T3* with peptide in K^+^ isolated from the other 10 spectra with PCA analysis (Figure 6).

From the above results, it is possible to confirm the successful performance of multivariate analysis in estimating the secondary structure of G-quadruplexes, as suggested by [35], and to support the above-described peptide G-quadruplex interaction.

### 3.4. Influence of Rhau25 Peptide on G4 Secondary Structure Stability

Photo-denaturation experiments as well as thermal denaturation experiments [45,46,47] were carried out to evaluate the folding stability of the G-quadruplex sequences in the presence and absence of the ***Rhau25*** peptide. 

**Figure 8:**
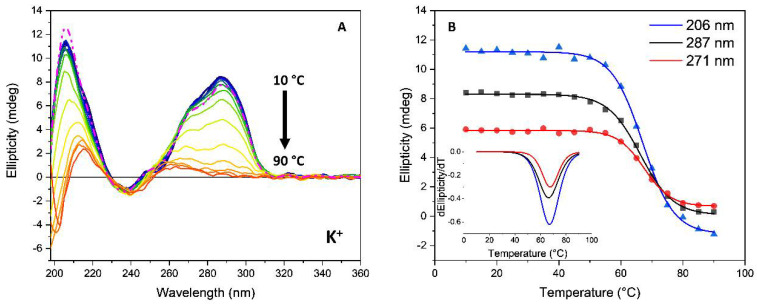
(**A**) SRCD spectra of *Htelo1* (13.5 µM) in 10 mM Tris-HCl buffer, pH 7.4, containing 70 mM potassium ions during the annealing process from 10 °C to 90 °C every 5 °C. The dashed line represents the oligonucleotide cooled to 20 °C after heating to 90 °C. The arrows represent the direction of the spectral change as the temperature increased. (**B**) SRCD-melting curves at 206 nm (blue), 271 nm (black) and 286 nm (red), respectively. Insert: Comparison of the corresponding first derivative of the SRCD as a function of temperature.

The SRCD spectra were recorded for the annealing process from 10 °C to 90 °C every 5 °C. To verify the presence of a two-state mechanism, F↔U, where F and U represent the folded and unfolded states, respectively, in the denaturation of G-quadruplex sequences, melting curves were constructed analyzing the CD intensity at two different wavelengths. If the two-state assumption is valid, the spectral data at the two wavelengths should be linearly correlated. The SRCD spectra as a function of temperature during the annealing process and the corresponding melting curve of *Htelo1* in the presence of potassium ions are illustrated in Figure 8 and the calculated T_m_ value in Table 2.

Only the *Htelo1* and *G3T3* oligomers, in the presence of potassium ions, as well as the *G3T3*-peptide complex in the presence of sodium ions, showed signs of two-state melting behavior (Figure 8, Figures S5 and S7 in Supplementary Materials) that enabled the calculation of the folded fraction at each temperature. On one side, the sloping of the baselines, which is often observed at low and/or high temperatures, has been fitted with straight lines by other authors to determine the folded fraction [48]. However, on the other side, this behavior could be interpreted as a two-transitions behavior, which shows a less stable conformation with T_m_ of about 28 °C and a more stable conformation with higher T_m_ (the latter indicated in Table 2).

**Figure 9:**
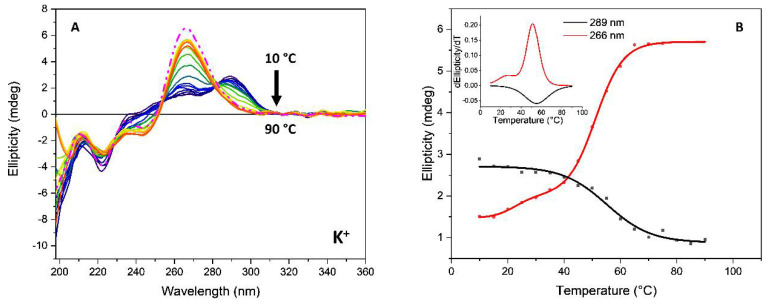
(**A**) Influence of temperature on the SRCD of *G3T3* (13.5 μM) with 2.2 eq. of ***Rhau25*** peptide in 10 mM Tris-HCl buffer, pH 7.4, 70 mM potassium ions. The temperature range was from 10 °C to 90 °C every 5 °C. The dashed line represents the oligonucleotide cooled to 20 °C after heating to 90 °C. The arrows represent the direction of the spectral change as the temperature is increased; (**B**) Plot of SRCD intensity versus temperature at 266 nm (red) and 289 nm (black). Insert: Comparison of the corresponding first derivative of the SRCD at 266 nm and 289 nm.

In terms of G4 folding, the annealing of *G3T3* with 2.2 equivalents of ***Rhau25*** peptide in K^+^ conducted in the same manner from 10 °C to 90 °C every 5 °C showed an increased content of parallel topology that reached the maximum content at 70 °C (Figure 9B) as revealed by the emergent positive band at 265 nm characteristic of the parallel topology (Figure 9A). The repeated thermal melting experiment from 10 °C to 90 °C every 10 °C of the previous annealed *G3T3* with ***Rhau25*** peptide (1:2.2) in K^+^ (Figure S9) revealed that the parallel topology was retained, being very stable at high temperatures without denaturing significantly even at 90 °C (Figure S8). It is important to note that the *G3T3* in K^+^ during and after annealing did not adopt the parallel topology (Figure S5, left). Indeed, melting experiment on the *G3T3*-***Rhau25*** complex performed on the G4 sample annealed in the presence of peptide shows that ***Rhau25*** strongly increased the stability of the complex, which did not achieve a fully denatured state at 90 °C (Figure S8).

**Figure 10:**
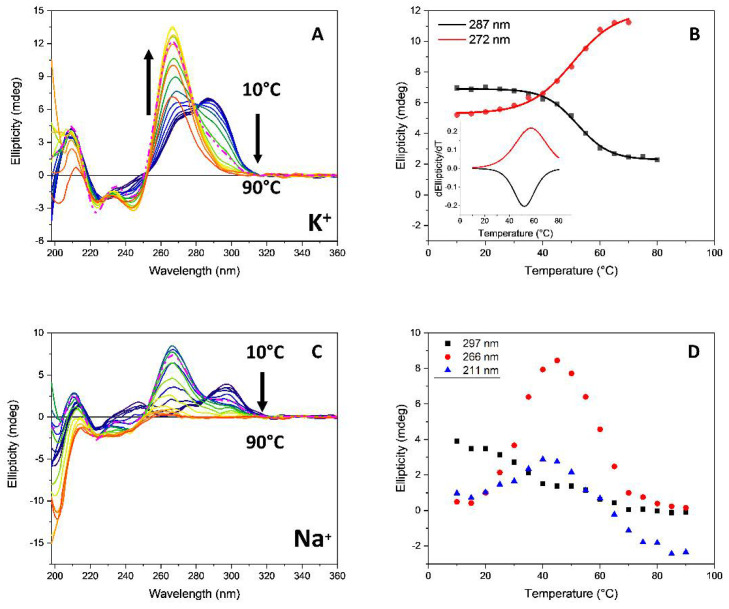
Thermal annealing of SRCD spectra of *Htelo1* (13.5 µM) with 2.2 eq. of ***Rhau25*** peptide in 10 mM Tris-HCl buffer, pH 7.4, containing 70 mM potassium ions (**A**) or sodium ions (**C**) from 10 °C to 90 °C every 5 °C. The dashed line represents the oligonucleotide cooled to 20 °C after heating to 90 °C. The arrows represent the direction of the signal change as the temperature is increased. (**B**) Plots of SRCD intensity versus temperature in K^+^ buffer at 272 nm (red) and 287 nm (black) with insert of the corresponding first derivatives. (**D**) Plots of SRCD versus temperature in Na^+^ buffer at 211 nm (blue), 266 nm (red), and 297 nm (black).

The stable parallel topology induced by annealing was also observed for the *Htelo1*-***Rhau25*** complex in the presence of potassium ions (Figure 10A). However, this was not the case in sodium ions (Figure 10C) where the increased formation of the parallel topology of *Htelo1* with 2.2 eq. ***Rhau25*** in Na^+^ was maximized up to 45 °C, as shown in Figure 10D, suggesting a possible denaturation above this temperature to unstacked single strand DNA. However, the slow conformational transition process may have hindered the accurate determination of the melting temperature of the complex. To verify this hypothesis, the SRCD spectrum of the *Htelo1*-***Rhau25*** complex, obtained by addition of 2.2 equivalent of ***Rhau25*** peptide in a single aliquot to the oligonucleotide rather than titrated with smaller aliquots, was monitored up to 90 min after the peptide addition (Figure S10). The CD melting experiment on *Htelo1* was repeated, allowing 5 h equilibration time after addition of the peptide, in order to assure the conformational transition would be completed (Figure S11). Therefore, a more accurate melting temperature could be determined (Table 2).

Multivariate statistical analysis of the SRCD spectra of *Htelo1-**Rhau25*** (1:2.2) in either sodium or potassium ions, and *G3T3-**Rhau25*** (1:2.2) solely in potassium ions, cooled to 20 °C after annealing at 90 °C showed that these G-quadruplex sequences exhibited a distinct behavior compared to all the other complexes evaluated in this study that are: *G3T3-**Rhau25*** (1:2.2) in Na^+^ and *T95-2T*-***Rhau25*** in both K^+^ and Na^+^ respectively (Figure S15).

**Figure 11:**
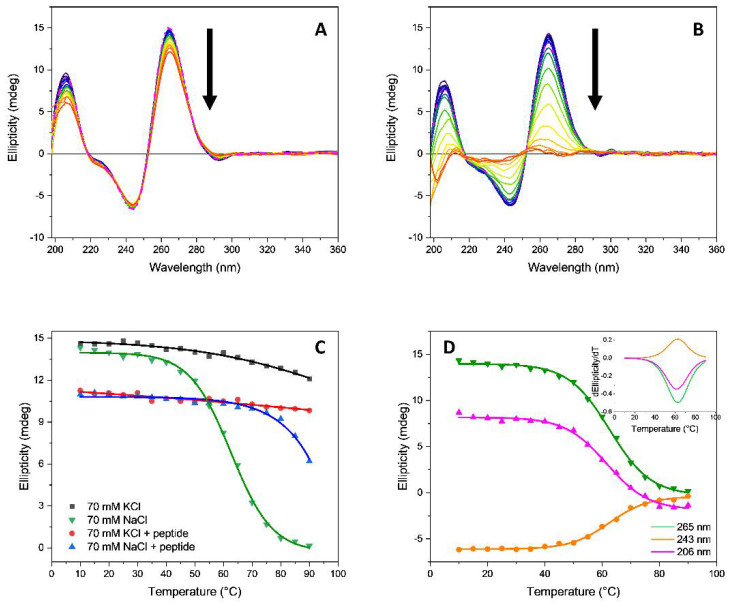
Influence of temperature on the SRCD spectra of *T95-2T* (13.5 µM) in 10 mM Tris-HCl buffer, pH 7.4, containing 70 mM (**A**) potassium or (**B**) sodium ions. The temperature varied from 10 to 90 °C. The dashed line represents the oligonucleotide cooled to 20 °C after heating to 90 °C. The arrows represent the direction of the spectral change as the temperature is increased; (**C**) SRCD-melting curves in sodium or potassium ions, and in the absence or presence of 2.2 equivalents of ***Rhau25*** peptide; (**D**) Melting curves of *T95-2T* in 10 mM Tris-HCl buffer, pH 7.4, containing 70 mM sodium ions at 206 nm (magenta), 243 nm (orange), and 265 nm (green) with insert for the corresponding first derivatives.

The *T95*-*2T* alone in potassium ions (Figure 11A) or when complexed with ***Rhau25*** peptide in the presence of sodium or potassium ions (Figure 5) was remarkably stable in the annealing process, retaining the parallel topology even at 90 °C. For this reason, it was not possible to determine their melting temperature T_m_ as the curves were not sigmoidal and the plateau was not reached at higher temperatures (Figure 11). On the contrary, in the presence of sodium ions, the *T95-2T* alone was able to reach a fully denatured state (Figure 11B,D), allowing the determination of the melting temperature (Table 2). This indicated that the ***Rhau25*** peptide stabilized the parallel topology of *T95*-*2T* in Na^+^ (Figure 5, right) unlike that in K^+^ where the peptide appeared to be not necessary (Figure 5, left). 

The analysis of the melting curves (Figure 11C) highlights how in K^+^ the parallel topology of *T95*-*2T* is more stable and thermally robust than in Na^+^, even when promoted by the ***Rhau25*** peptide (Figure 11B). 

Apart from the G-quadruplex/peptide complexes characterized by a conformational conversion (indicated with * in Table 2), the CD spectra of not annealed and annealed at 20 °C were almost superimposable, indicating that the G-quadruplexes with or without the peptide were reversible. On the contrary, the conformational conversion induced upon heating was also retained upon cooling, indicating that the new conformation was the preferred one.

**Figure 12:**
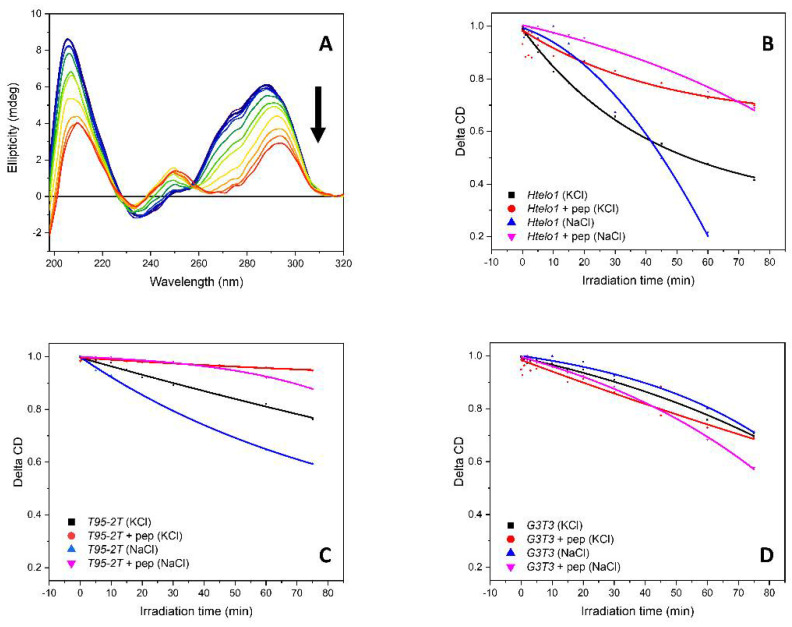
(**A**) CD spectra G-quadruplex sequence (as example, *Htelo1* 13.5 µM in 10 mM Tris-HCl buffer, pH 7.4, in presence of 70 mM potassium ions) as a function of irradiation time with the arrow representing the direction of the spectral change as irradiation time is increased. Normalized rates of denaturation of G-quadruplex-forming sequences with and without the ***Rhau25*** peptide in 70 mM potassium or sodium ions; (**B**) for *Htelo1*; (**C**) for *T95-2T*; (**D**) for *G3T3*.

Another behavior towards perturbations that is informative and useful to determine is the UV-photo-stability of the G4-sequences in presence or not of the ***Rhau25*** peptide (1:2.2 molar ratio). The CD spectra were recorded as a function of irradiating time with either a UV-C lamp or the synchrotron radiation (Figure 12, Figures S17–S21). Under these conditions, the observed conformational denaturation has been attributed to the action of reactive oxygen species (ROS) generated by the photolysis of water molecules, as recently demonstrated using a positive fluorophore probe [49].

In terms of relative stability among the three sequences *G3T3*, *Htelo1* and *T95-2T* investigated under the four conditions of 70 mM K^+^, 70 mM Na^+^, with and without 2.2 eq. of ***Rhau25*** peptide in K^+^ or Na^+^, the UV denaturation curves in Figure 12B–D indicated that the sequence *T95-2T* with 2.2 ***Rhau25*** peptide in K^+^ (red lines) was the most UV stable followed by both *G3T3* and *Htelo1*. This order was maintained with the peptide in Na^+^ (blue lines), though these were less stable than those with the peptide in K^+^ (red lines). Without the peptide in K^+^ (black lines), both *T95-2T* and *G3T3* appeared to have similar UV stability followed by the less stable *Htelo1*. On the other hand, without the peptide in Na^+^ (magenta lines), *G3T3* was the most stable followed by *T95-2T* with *Htelo1* the last one.

In terms of relative stability for each G-quadruplex sequence studied under the four conditions of 70 mM K^+^, 70 mM Na^+^, with and without 2.2 eq. of ***Rhau25*** peptide in K^+^ or Na^+^, the UV denaturation curves in Figure 12B–D indicated that the stabilities were more similar for *G3T3* than *T95-2T* than *Htelo1* sequences.

In terms of the effect of the ***Rhau25*** peptide on the UV stability of the induced G-quadruplex topology, the *G3T3* sequence was less UV stable, suggesting that the addition of the peptide induced a G4 structure more prone to the denaturing effects induced by ROS.

## 4. Conclusions

In this study, circular dichroism spectroscopy was utilized to monitor the structure and interaction with a small peptide, containing the sequence 52–75 of the N-terminal domain of the human RHAU helicases, of three G-quadruplex-forming sequences able to adopt different structure topologies, extending early studies by other authors on the properties of RHAU derived peptides.

We have reported experimental evidence that not only does the ***Rhau25*** peptide specifically recognize parallel G-quadruplex structure, in the *T95-2T* sequence, with high affinity, but also its interaction with antiparallel or hybrid *G3T3* and *Htelo1* structures induced a conformational conversion of the G4-strucure to the parallel topology. This transition is favored in the presence of sodium ions coordinated within the plane of G4 structure. It is also observed in presence of highly stabilizing potassium ions after annealing the peptide/G4 complexes.

The pharmaceutical relevance of these findings lies in the widespread presence in regions of the genome of G-quadruplex-forming sequences, making these structures a promising drug target not only in the discovery of anticancer but also antiviral drugs, especially against viruses that exhibit latency. Binding to specific G-quadruplex topologies, as the ***Rhau25*** peptide does, will also be an important aspect of drug design. With numerous G-quadruplexes present in the human genome, binding to one particular structure will be important in targeting transcription of individual genes.

In addition, to directly inhibit the transcriptional mechanism by stabilizing G-quadruplexes, the use of peptide-based drugs can also block the interaction between nucleic acids and specific proteins essential to allow G-quadruplexes to perform their function. Furthermore, the properties of peptides can improve cellular permeation and targeting, increasing the ability to target specific organelles, a feature not always present in small molecules.

Overall, the data presented here confirms the usefulness of multivariate analysis in assigning the G4 secondary structure on the basis of CD spectral profile that can be further used to understand G-quadruplex behavior, inspiring new therapeutic possibilities.

## Figures and Tables

**Table 1:** *K_d_* values calculated for each oligonucleotide strand in the presence of ***Rhau25*** peptide.

	*K_d_* (µM)	
*G4 sequence*	KCl	NaCl
*G3T3*	12.2 ± 0.8	14 ± 0.3
*Htelo1*	27 ± 1	30 ± 8
*T95-2T*	13.5 ± 0.5	13.9 ± 1

**Table 2:** Melting temperature values for the G-quadruplex sequences with and without the ***Rhau25*** peptide.

	Melting Temperature T_m_ (°C)	
Sample	KCl	NaCl
*G3T3* alone	69 ± 0.3	51 ± 0.4
*G3T3* + peptide	n.d. *	69 ± 0.3
*Htelo1* alone	66 ± 0.3	53 ± 0.3
*Htelo1* + peptide	n.d. *	59 ± 0.4 *^,#^
*T95-2T* alone	>90	61 ± 0.2
*T95-2T* + peptide	>90	>90

n.d. not determinable because of high stability of the structure. * observed conformational conversion. ^#^ determined after 5 h equilibration time.

## Supplementary Materials

The following are available online at https://www.mdpi.com/article/10.3390/pharmaceutics13081104/s1, Figure S1: LC-ESI-MS characterization of the ***Rhau25*** peptide; Figure S2: Interaction of the ***Rhau25*** peptide with ctDNA; Figure S3: CD titration curves of the investigated G4-DNA strands with up to 2.2 eq. ***Rhau25*** peptide; Figure S4: Calculation of the ***Rhau25*** peptide conformation when interacting with the investigated G4 sequences; Figure S5: CD melting experiment on *G3T3* in KCl buffer; Figure S6: CD melting experiment on *G3T3* in NaCl buffer; Figure S7: CD melting experiment on *G3T3* + 2.2 molar equivalents of ***Rhau25*** in NaCl buffer; Figure S8: CD melting experiment on *G3T3* + 2.2 molar equivalents of ***Rhau25*** in KCl buffer after annealing of the G4 sequence in presence of peptide; Figure S9: CD annealing and melting experiments on *G3T3* + 2.2 molar equivalents of ***Rhau25*** in KCl buffer; Figure S10: Conformational conversion of the *Htelo1* quadruplex by the ***Rhau25*** peptide in NaCl buffer monitored for up to 90 min; Figure S11: CD melting experiment on *Htelo1* + 2.2 molar equivalents of ***Rhau25*** in NaCl buffer after 5 h equilibration time; Figure S12: CD melting experiment on *Htelo1* in NaCl buffer; Figure S13: CD melting experiment on *T95-2T* + 2.2 molar equivalents of ***Rhau25*** in KCl buffer; Figure S14: CD melting experiment on *T95-2T* + 2.2 molar equivalents of ***Rhau25*** in NaCl buffer; Figure S15: PCA plot identifying the main clusters indicative of G4 conformation, including data recorded at 20 °C after the heating and cooling experiment for the samples that showed a conformational change; Figure S16: Hierarchical cluster analysis plot identifying the main clusters indicative of G4 conformation including data recorded at 20 °C after the heating and cooling experiment for the samples that showed a conformational change; Figure S17: UV denaturation experiment of *G3T3* alone or in presence of ***Rhau25*** in KCl buffer; Figure S18: UV denaturation experiment of *G3T3* alone or in presence of ***Rhau25*** in NaCl buffer; Figure S19: UV denaturation experiment of *Htelo1* alone or in presence of ***Rhau25*** in NaCl buffer; Figure S20: UV denaturation experiment of *T95-2T* alone or in presence of ***Rhau25*** in KCl buffer; Figure S21: UV denaturation experiment of *T95-2T* alone or in presence of ***Rhau25*** in NaCl buffer.

## References

[1] Tateishi-Karimata H., Sugimoto N. (2020). Chemical biology of non-canonical structures of nucleic acids for therapeutic applications. Chem. Commun..

[2] Rhodes D., Lipps H.J. (2015). G-quadruplexes and their regulatory roles in biology. Nucleic Acids Res..

[3] Ruggiero E., Richter S.N. (2020). Viral G-quadruplexes: New frontiers in virus pathogenesis and antiviral therapy. Annu. Rep. Med. Chem..

[4] Yadav P., Kim N., Kumari M., Verma S., Sharma T.K., Yadav V., Kumar A. (2021). G-Quadruplex Structures in Bacteria—Biological Relevance and Potential as Antimicrobial Target. J. Bacteriol..

[5] Dumetz F., Merrick C.J. (2019). Parasitic Protozoa: Unusual Roles for G-Quadruplexes in Early-Diverging Eukaryotes. Molecules.

[6] Amrane S., Kerkour A., Bedrat A., Vialet B., Andreola M.-L., Mergny J.-L. (2014). Topology of a DNA G-Quadruplex Structure Formed in the HIV-1 Promoter: A Potential Target for Anti-HIV Drug Development. J. Am. Chem. Soc..

[7] Ruggiero E., Tassinari M., Perrone R., Nadai M., Richter S.N. (2019). Stable and Conserved G-Quadruplexes in the Long Terminal Repeat Promoter of Retroviruses. ACS Infect. Dis..

[8] Virgilio A., Esposito V., Tassinari M., Nadai M., Richter S.N., Galeone A. (2020). Novel monomolecular derivatives of the anti-HIV-1 G-quadruplex-forming Hotoda’s aptamer containing inversion of polarity sites. Eur. J. Med. Chem..

[9] Ruggiero E., Richter S.N. (2018). G-quadruplexes and G-quadruplex ligands: Targets and tools in antiviral therapy. Nucleic Acids Res..

[10] Murat P., Zhong J., Lekieffre L., Cowieson N.P., Clancy J.L., Preiss T., Balasubramanian S., Khanna R., Tellam J. (2014). G-quadruplexes regulate Epstein-Barr virus–encoded nuclear antigen 1 mRNA translation. Nat. Chem. Biol..

[11] Marušič M., Plavec J. (2019). Towards Understanding of Polymorphism of the G-rich Region of Human Papillomavirus Type 52. Molecules.

[12] Majee P., Pattnaik A., Sahoo B.R., Shankar U., Pattnaik A.K., Kumar A., Nayak D. (2021). Inhibition of Zika virus replication by G-quadruplex-binding ligands. Mol. Ther.-Nucl. Acids.

[13] Ji D., Juhas M., Tsang C.M., Kwok C.K., Li Y., Zhang Y. (2021). Discovery of G-quadruplex-forming sequences in SARS-CoV-2. Brief. Bioinform..

[14] Maizels N., Gray L.T. (2013). The G4 genome. PLoS Genet..

[15] Zakian V.A. (2012). Telomeres: The beginnings and ends of eukaryotic chromosomes. Exp. Cell Res..

[16] Bochman M.L., Paeschke K., Zakian V.A. (2012). DNA secondary structures: Stability and function of G-quadruplex structures. Nat. Rev. Genet..

[17] De Cian A., Lacroix L., Douarre C., Temime-Smaali N., Trentesaux C., Riou J.F., Mergny J.L. (2008). Targeting telomeres and telomerase. Biochimie.

[18] Hurley L.H. (2002). DNA and its associated processes as targets for cancer therapy. Nat. Rev. Cancer.

[19] Yaku H., Fujimoto T., Murashima T., Miyoshi D., Sugimoto N. (2012). Phthalocyanines: A new class of G-quadruplex-ligands with many potential applications. Chem. Commun..

[20] Cavallari M., Garbesi A., Di Felice R. (2009). Porphyrin intercalation in G4-DNA quadruplexes by molecular dynamics simulations. J. Phys. Chem. B.

[21] Sissi C., Gatto B., Palumbo M. (2011). The evolving world of protein-G-quadruplex recognition: A medicinal chemist’s perspective. Biochimie.

[22] Tucker W.O., Shum K.T., Tanner J.A. (2012). G-quadruplex DNA aptamers and their ligands: Structure, function and application. Curr. Pharm. Des..

[23] Usui K., Okada A. (2014). Peptides Targeting G-Quadruplex Structures. Chemical Biology of Nucleic Acids: Fundamentals and Clinical Applications.

[24] Liu K.C., Röder K., Mayer C., Adhikari S., Wales D.J., Balasubramanian S. (2020). Affinity-Selected Bicyclic Peptide G-Quadruplex Ligands Mimic a Protein-like Binding Mechanism. J. Am. Chem. Soc..

[25] Biswas S., Samui S., Das A.K., Pasadi S., Muniyappa K., Naskar J. (2020). Targeting G-quadruplex DNA with synthetic dendritic peptide: Modulation of the proliferation of human cancer cells. RSC Adv..

[26] Huang Z.L., Dai J., Luo W.H., Wang X.G., Tan J.H., Chen S.B., Huang Z.S. (2018). Identification of G-Quadruplex-Binding Protein from the Exploration of RGG Motif/G-Quadruplex Interactions. J. Am. Chem. Soc..

[27] Tyagi S., Saxena S., Kundu N., Sharma T., Chakraborty A., Kaur S., Miyoshi D., Shankaraswamy J. (2019). Selective recognition of human telomeric G-quadruplex with designed peptide via hydrogen bonding followed by base stacking interactions. RSC Adv..

[28] Heddi B., Cheong V.V., Martadinata H., Phan A.T. (2015). Insights into G-quadruplex specific recognition by the DEAH-box helicase RHAU: Solution structure of a peptide-quadruplex complex. PNAS.

[29] Ariyo E.O., Booy E.P., Patel T.R., Dzananovic E., McRae E.K., Meier M., McEleney K., Stetefeld J., McKenna S.A. (2015). Biophysical Characterization of G-Quadruplex Recognition in the PITX1 mRNA by the Specificity Domain of the Helicase RHAU. PLoS ONE.

[30] Chen M.C., Tippana R., Demeshkina N.A., Murat P., Balasubramanian S., Myong S., Ferré-D’Amaré A.R. (2018). Structural basis of G-quadruplex unfolding by the DEAH/RHA helicase DHX36. Nature.

[31] Meier M., Patel T.R., Booy E.P., Marushchak O., Okun N., Deo S., Howard R., McEleney K., Harding S.E., Stetefeld J. (2013). Binding of G-quadruplexes to the N-terminal recognition domain of the RNA helicase associated with AU-rich element (RHAU). J. Biol. Chem..

[32] Yaneva M.Y., Cheong V.V., Cheng J.K., Lim K.W., Phan A.T. (2020). Stapling a G-quadruplex specific peptide. Biochem. Byophys. Res. Comm..

[33] Bhattacharyya D., Mirihana Arachchilage G., Basu S. (2016). Metal Cations in G-Quadruplex Folding and Stability. Front. Chem..

[34] Behrendt R., White P., Offer J. (2016). Advances in Fmoc solid-phase peptide synthesis. J. Pep. Sci..

[35] Del Villar-Guerra R., Trent J.O., Chaires J.B. (2018). G-Quadruplex Secondary Structure Obtained from Circular Dichroism Spectroscopy. Angew. Chem. Int. Ed. Engl..

[36] Vorlíčková M., Kejnovská I., Bednářová K., Renčiuk D., Kypr J. (2012). Circular dichroism spectroscopy of DNA: From duplexes to quadruplexes. Chirality.

[37] Vorlíčková M., Kejnovská I., Sagi J., Renčiuk D., Bednářová K., Motlová J., Kypr J. (2012). Circular dichroism and guanine quadruplexes. Methods (San Diego Calif.).

[38] Randazzo A., Spada G.P., Da Silva M.W. (2013). Circular dichroism of quadruplex structures. Top. Curr. Chem..

[39] Lim K.W., Ng V.C., Martín-Pintado N., Heddi B., Phan A.T. (2013). Structure of the human telomere in Na+ solution: An antiparallel (2 + 2) G-quadruplex scaffold reveals additional diversity. Nucleic Acids Res..

[40] Lim K.W., Amrane S., Bouaziz S., Xu W., Mu Y., Patel D.J., Luu K.N., Phan A.T. (2009). Structure of the Human Telomere in K+ Solution: A Stable Basket-Type G-Quadruplex with Only Two G-Tetrad Layers. J. Am. Chem. Soc..

[41] Islam B., Stadlbauer P., Krepl M., Havrila M., Haider S., Sponer J. (2018). Structural Dynamics of Lateral and Diagonal Loops of Human Telomeric G-Quadruplexes in Extended MD Simulations. J. Chem. Theory Comput..

[42] Villani G. (2018). Quantum Mechanical Investigation of the G-Quadruplex Systems of Human Telomere. ACS Omega.

[43] Zhang X., Xu C.-X., Di Felice R., Sponer J., Islam B., Stadlbauer P., Ding Y., Mao L., Mao Z.-W., Qin P.Z. (2016). Conformations of Human Telomeric G-Quadruplex Studied Using a Nucleotide-Independent Nitroxide Label. Biochemistry.

[44] Hill A.V. (1910). The possible effects of the aggregation of the molecules of hæmoglobin on its dissociation curves. J. Physiol..

[45] Di Gaspero M., Ruzza P., Hussain R., Honisch C., Biondi B., Siligardi G., Marangon M., Curioni A., Vincenzi S. (2020). The Secondary Structure of a Major Wine Protein is Modified upon Interaction with Polyphenols. Molecules.

[46] Honisch C., Hussain R., Siligardi G., Ruzza P. (2020). Influence of small molecules on the photo-stability of water soluble porcine lens proteins. Chirality.

[47] Hussain R., Longo E., Siligardi G. (2018). UV-Denaturation Assay to Assess Protein Photostability and Ligand-Binding Interactions Using the High Photon Flux of Diamond B23 Beamline for SRCD. Molecules.

[48] Rachwal P.A., Fox K.R. (2007). Quadruplex melting. Methods (San Diego Calif.).

[49] Ruzza P., Honisch C., Hussain R., Siligardi G. (2021). Free Radicals and ROS Induce Protein Denaturation by UV Photostability Assay. Int. J. Mol. Sci..

