# Supplementary material for: Interaction of a Short Peptide with G-Quadruplex-Forming Sequences: An SRCD and CD Study
Source: Pharmaceutics. 2021 Jul 21;13(8):1104. doi: 10.3390/pharmaceutics13081104 (PMC8401852; doi:10.3390/pharmaceutics13081104)
Supplement: Supplementary file 1 [file pharmaceutics-13-01104-s001.zip › pharmaceutics-1262305-supplementary.pdf]

pharmaceutics

MDPI

Supplementary Materials: Interaction of a Short Peptide with G-Quadruplex-Forming Sequences: An SRCD and CD Study

Claudia Honisch, Eugenio Ragazzi, Rohanah Hussain, John Brazier, Giuliano Siligardi and Paolo Ruzza

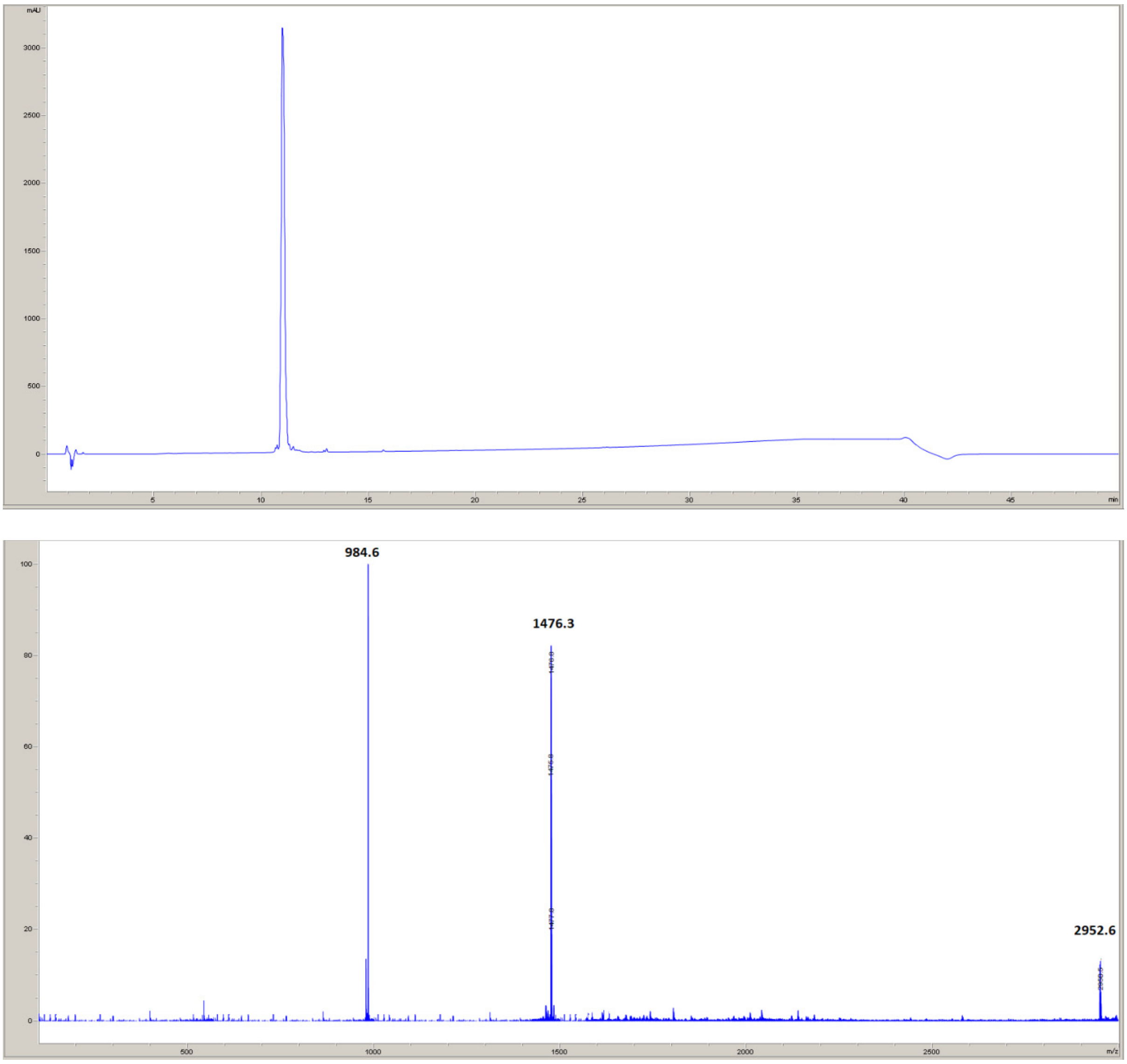

Figure S1. LC-ESI-MS characterisation of ***Rhau25*** peptide. Ac-*Ser-Met-His-Pro-Gly-His-Leu-Lys-Gly-Arg-Glu-Ile-Gly-Met-Trp-Tyr-Ala-Lys-Lys-Gln-Gly-Gln-Lys-Asn-Lys*-NH_2. The calculated mass was 2951.41 Da, the determined mass $[M+H]^+$ resulted to be 2952.55 m/z, $[M+2H]^{2+}$ was 1476.30 m/z and $[M+3H]^{3+}$ was 984.6 m/z.

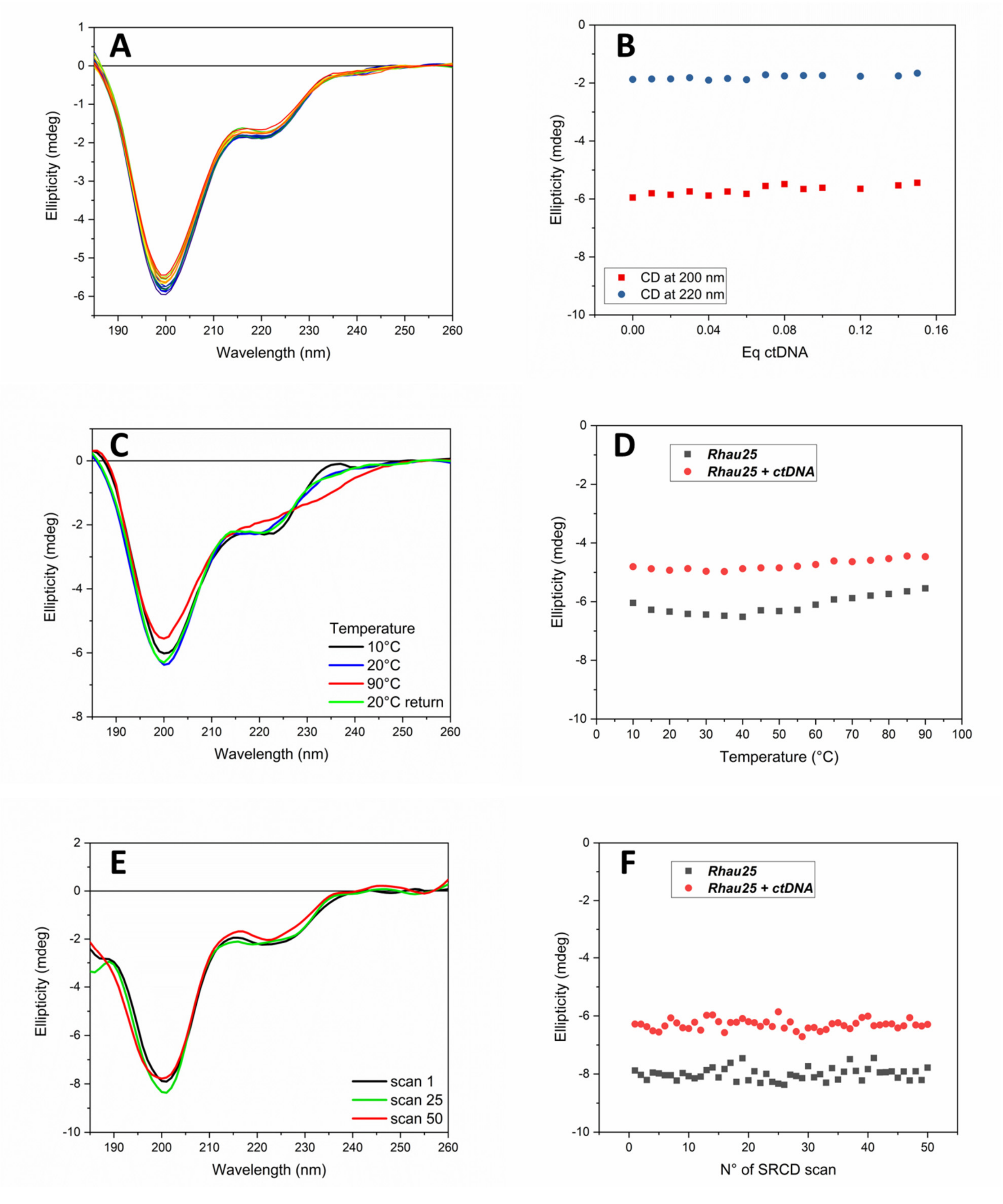

Figure S2. (**A**) CD spectra of ***Rhau25*** peptide (30.5 μM) recorded in a 0.1 cm pathlength suprasil quartz cuvette in absence (black line) and in presence of increasing amounts of ctDNA, up to 0.15 molar equivalents, in 10 mM Tris-HCl buffer, pH 7.4; (**B**) Ellipticity values at 200 nm (red) and 220 nm (blue) as a function of ctDNA added; (**C**) Influence of temperature on the CD spectrum of ***Rhau25*** in 10 mM Tris-HCl buffer, pH 7.4; (**D**) Ellipticity values at 200 nm as a function of temperature for ***Rhau25*** peptide alone (black) or in presence of 15 molar equivalents of ctDNA (red). (**E**) 50 consecuive SRCD spectra of ***Rhau25*** peptide (154 μM) recorded in a 0.02 cm suprasil quartz cuvette; (**F**) Ellipticity values at 200 nm as a function of SRCD scans. Spectra were acquired on module A of beamline B23 at Diamond Light Source syncrotron at 39 nm/min, Data Integration Time 1s, data pitch 0.5 nm, bandwidth 1 nm.

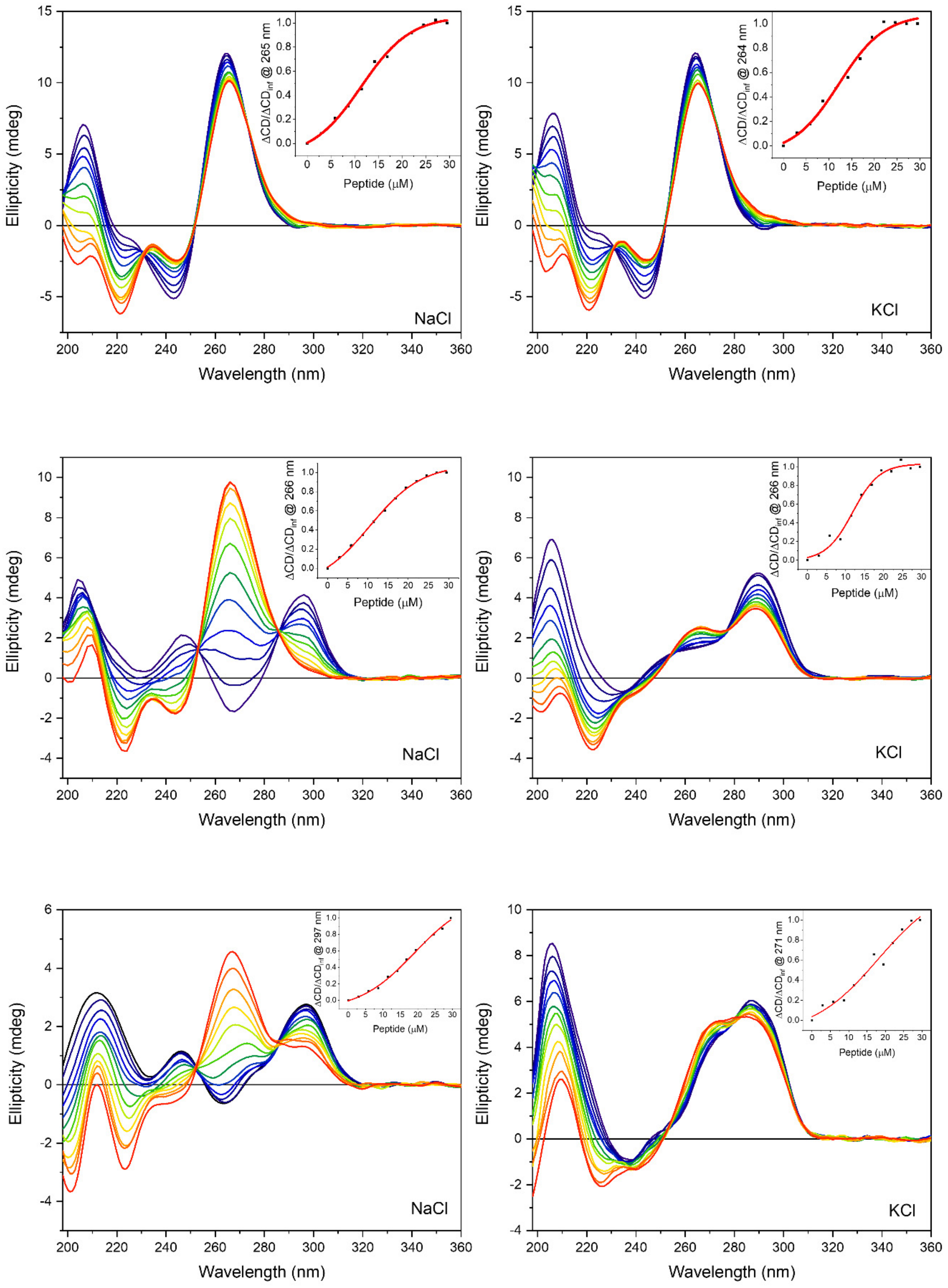

Figure S3. CD spectra and relative titration curves (inserts) of the investigated G4-DNA strands, (upper row) *T95-2T*, (center) *G3T3*, and (lower row) *Htelo1*, 13.5 μM in10 mM Tris-HCl containing 70 mM of either sodium or potassium ions (indicated), with up to 2.2 eq. ***Rhau25*** peptide. Curves are built fitting the change in elleipticity at 265 nm with a non-linear regression equation (Hill1 Fit in OriginPro2018, OriginLab Corporation).

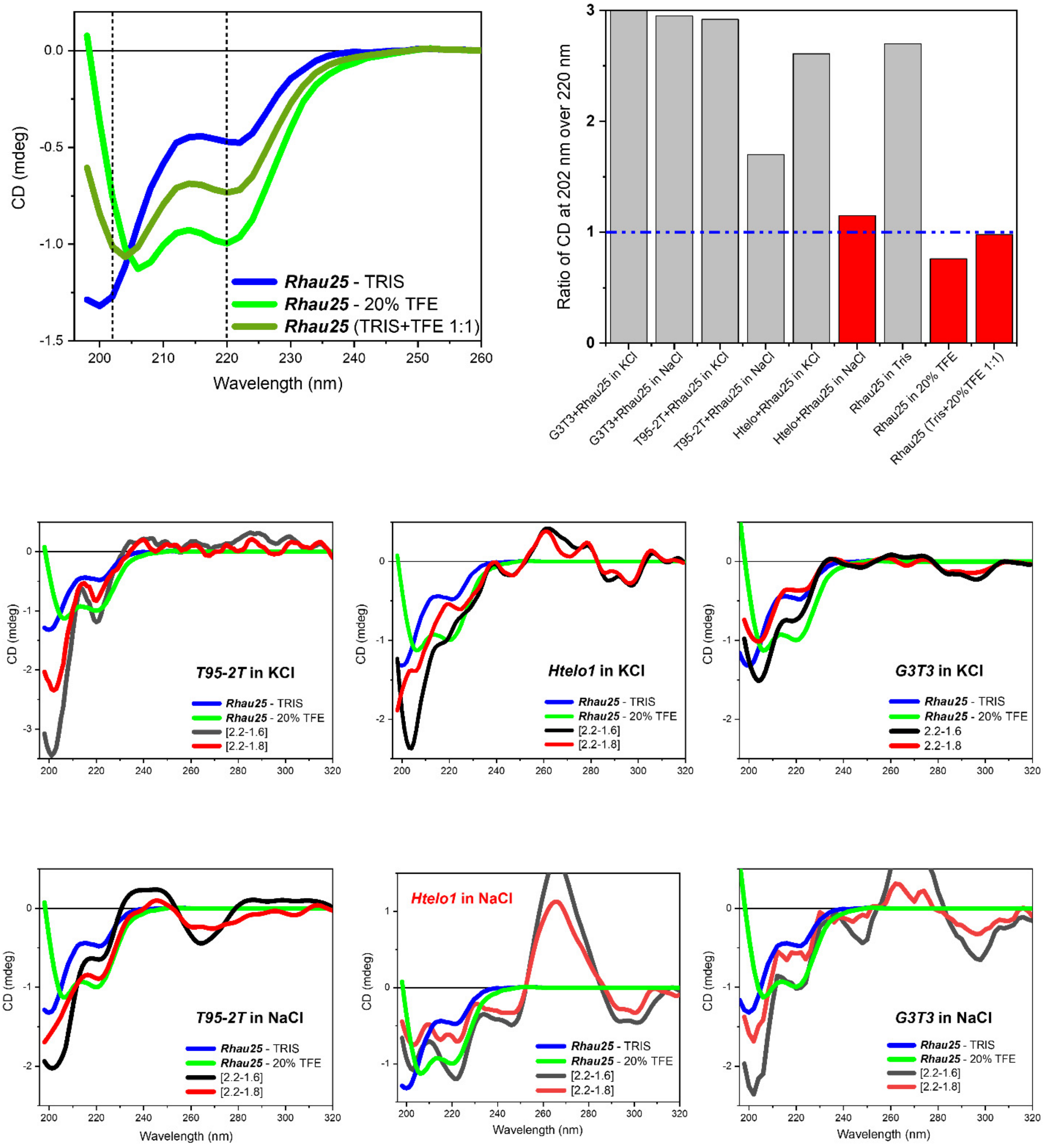

Figure S4. Calculated CD spectra subtracting the spectrum of the G-quadruplex bound to the ligand peptide in the molar ratio (1:1.8) to that of the complex with molar ratio (1:2.2). Ratios at about 1 and below suggest that the bound conformation of the peptide to *Htelo* in NaCl is similar to that of the peptide in 20% TFE indicative of a high α-helical content. In all other complexes, the bound ***Rhau25*** peptide appears to retain a similar conformation observed in Tris-HCl that is largely unordered.

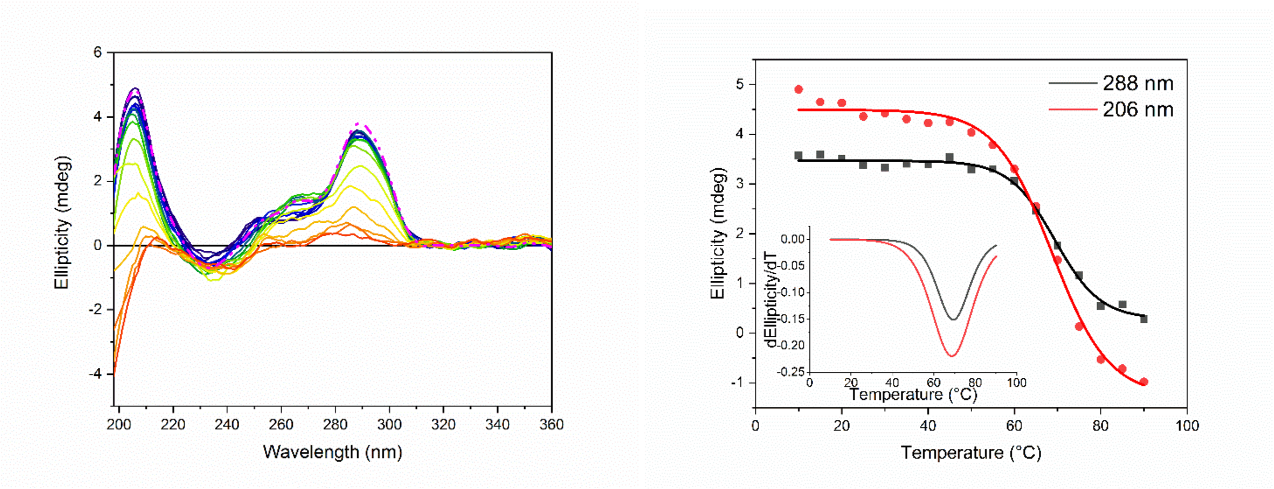

Figure S5. (**left**) Influence of temperature on the CD spectrum of *G3T3* (13.5 µM) in 10 mM Tris-HCl buffer, pH 7.4, added of 70 mM KCl. The temperature varied from 10 to 90°C. The dashed line represents the oligonucleotide cooled to 20°C after heating to 90°C. Spectra were acquired on module A of beamline B23 at Diamond Light Source syncrotron in a 0.1 cm pathlength quartz cuvette at 39 nm/min, DIT 1s, data pitch 0.5 nm, bandwidth 1 nm, in the 10-90°C range with steps of 5°C and allowing 2 minutes equilibration. (**right**) CD-melting curves at different wavelengths (indicated). Insert: comparison of the corresponding first derivative of the signal with respect to temperature at the indicated wavelength.

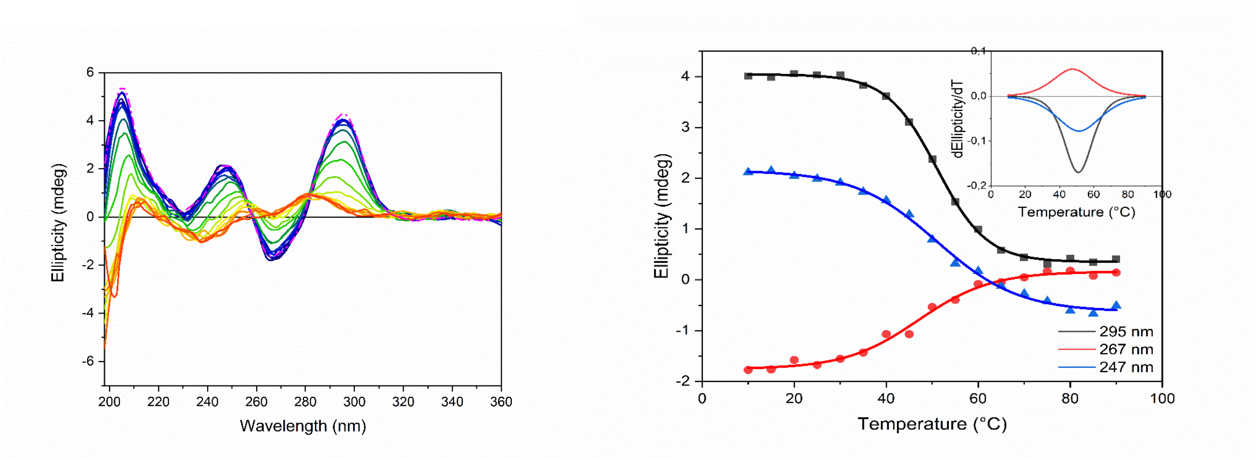

Figure S6. (**left**) Influence of temperature on the CD spectrum of *G3T3* (13.5 µM) in 10 mM Tris-HCl buffer, pH 7.4, 70 mM NaCl. The temperature varied from 10°C to 90°C every 5°C. The dashed line represents the oligonucleotide cooled to 20°C after heating to 90°C. Spectra were acquired on module A of beamline B23 at Diamond Light Source syncrotron in a 0.1 cm pathlength quartz cuvette at 39 nm/min, DIT 1s, data pitch 0.5 nm, bandwidth 1 nm, in the 10-90°C range with steps of 5°C and allowing 2 minutes equilibration. (**right**) CD-melting curves at 247 nm (blue), 267 nm (red) and 295 nm (black) with insert for the comparison of the corresponding first derivatives.

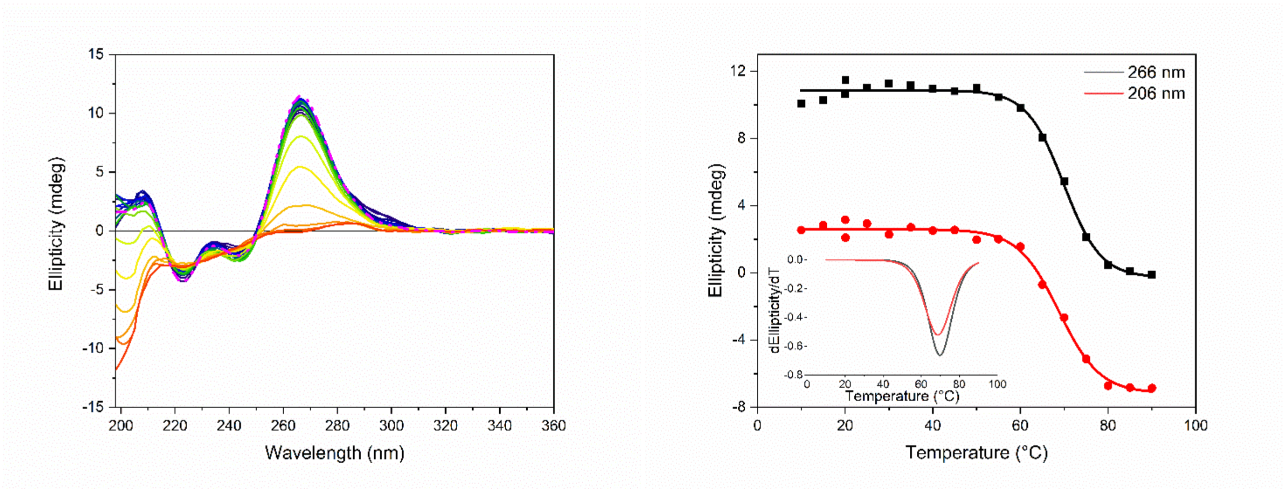

Figure S7. (**left**) Influence of temperature on the CD spectrum of *G3T3* (13.5µM) in presence of 2.2 molar equivalents of ***Rhau25*** in 10mM Tris-HCl buffer, pH 7.4, added of 70 mM NaCl. The temperature varied from 10°C to 90°C every 5°C. The dashed line represents the oligonucleotide cooled to 20°C after heating to 90°C. Spectra were acquired on module A of beamline B23 at Diamond Light Source syncrotron in a 0.1 cm pathlength quartz cuvette at 39 nm/min, DIT 1s, data pitch 0.5 nm, bandwidth 1 nm, in the 10-90°C range with steps of 5°C and allowing 2 minutes equilibration. (**right**) CD-melting curves at 206nm (red) and 266nm (black) with insert for the comparison of the corresponding first derivatives.

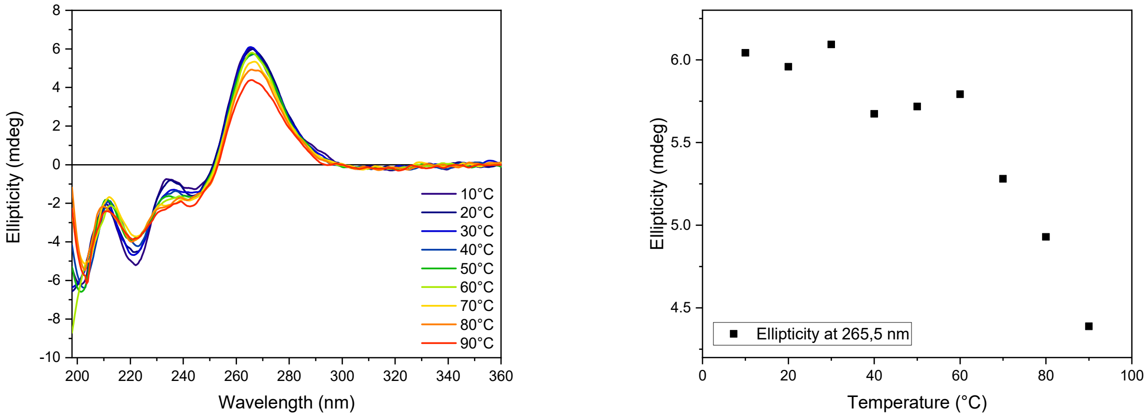

Figure S8. (**left**) Influence of temperature on the CD spectrum of *G3T3* (13.5 μM) in presence of 2.2 molar equivalents of ***Rhau25*** in 10 mM Tris-HCl buffer, pH 7.4, added of 70 mM KCl. The CD melting was recorded in the 10°C to 90°C range every 10°C after annealing of the *G3T3* in presence of the peptide. Spectra were acquired on module A of beamline B23 at Diamond Light Source syncrotron in a 0.1 cm pathlength quartz cuvette at 39 nm/min, DIT 1s, data pitch 0.5 nm, bandwidth 1 nm, in the 10-90°C range with steps of 10°C and allowing 2 minutes equilibration. (**right**) CD-melting curves at 265.5 nm.

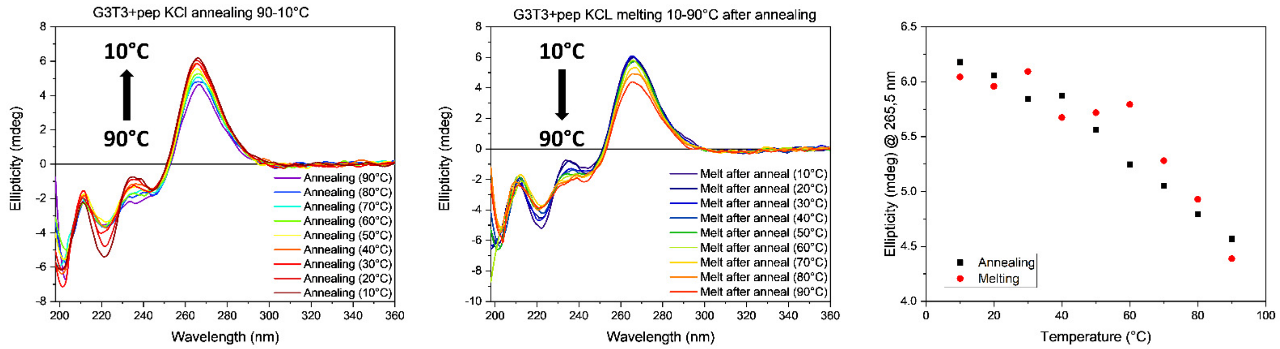

Figure S9. (**left**) Annealing of *G3T3* (13.5 μM) in presence of 2.2 molar equivalents of ***Rhau25*** in 10 mM TRIS-HCl buffer, pH 7.4, added of 70 mM KCl. The CD spectra were recorded in the 90°C to 10°C range every 10°C on module A of beamline B23 at Diamond Light Source syncrotron in a 0.1 cm pathlength quartz cuvette at 39 nm/min, DIT 1s, data pitch 0.5 nm, bandwidth 1 nm; (**center**) Melting of *G3T3* (13.5 μM) in presence of 2.2 molar equivalents of ***Rhau25*** in 10mM Tris-HCl buffer, pH 7.4, added of 70 mM KCl. The CD spectra were recorded in the 10°C to 90°C range every 10°C on module A of beamline B23 at Diamond Light Source syncrotron in a 0.1 cm pathlength quartz cuvette at 39 nm/min, DIT 1s, data pitch 0.5 nm, bandwidth 1 nm; (**right**) CD-annealing (black) and melting (red) curves at 265.5 nm.

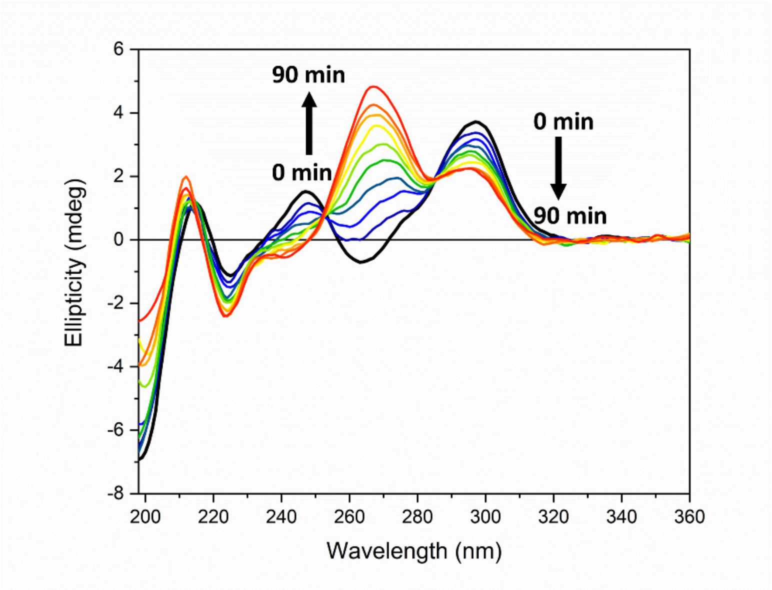

Figure S10. Conformational conversion of the *HTelo1* quadruplex by ***Rhau25*** peptide assessed by CD spectroscopy. Quadruplex was prepared in 10 mM Tris-HCl buffer, pH 7.4, containing 70 mM Na^+, and added of 2.2 eq. of ***Rhau25*** peptide in one aliquot. Oligonucleotide concentration was 13.5 µM. The arrows represent the direction of the signal change as the time is increased. Spectra were recorded on a Jasco J1500 spectrometer, scan speed 100 nm/min, DIT 1s, data pitch 0.5 nm, bandwidth 2 nm, in a 1 mm pathlength quartz cuvette at 25°C.

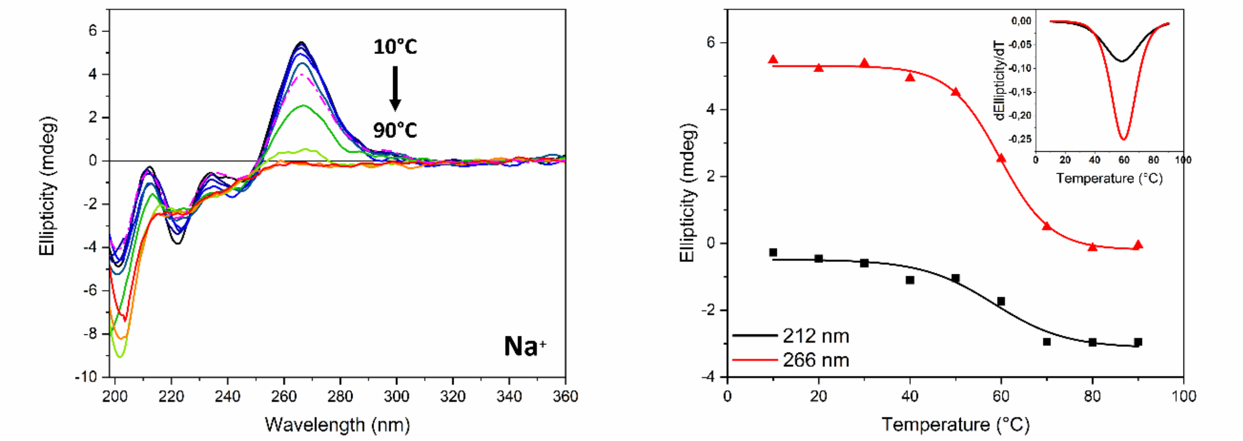

Figure S11. (A) Influence of temperature on the CD spectrum of *HTelo1* sequence in 10 mM Tris-HCl buffer, pH 7.4, containing 70 mM Na^+ in presence of 2.2 eq. of ***Rhau25*** peptide. SRCD spectra were recorded 5 hours after peptide addition in one aliquot. The temperature varied from 10 to 90°C. The dashed line represents the oligonucleotide cooled to 20°C after heating to 90°C. Oligonucleotide concentration was 13.5 µM. The arrows represent the direction of the signal change as the temperature is increased. **(B)** CD-melting curves at different wavelengths (indicated). Insert: comparison of the corresponding first derivative of the signal with respect to temperature at the indicated wavelength. Spectra were recorded on module A, Beamline B23, Dimond Light Source Ltd., scan speed 39 nm/min, DIT 1s, data pitch 0.5 nm, bandwidth 1 nm, in a 1 mm pathlength quartz cuvette.

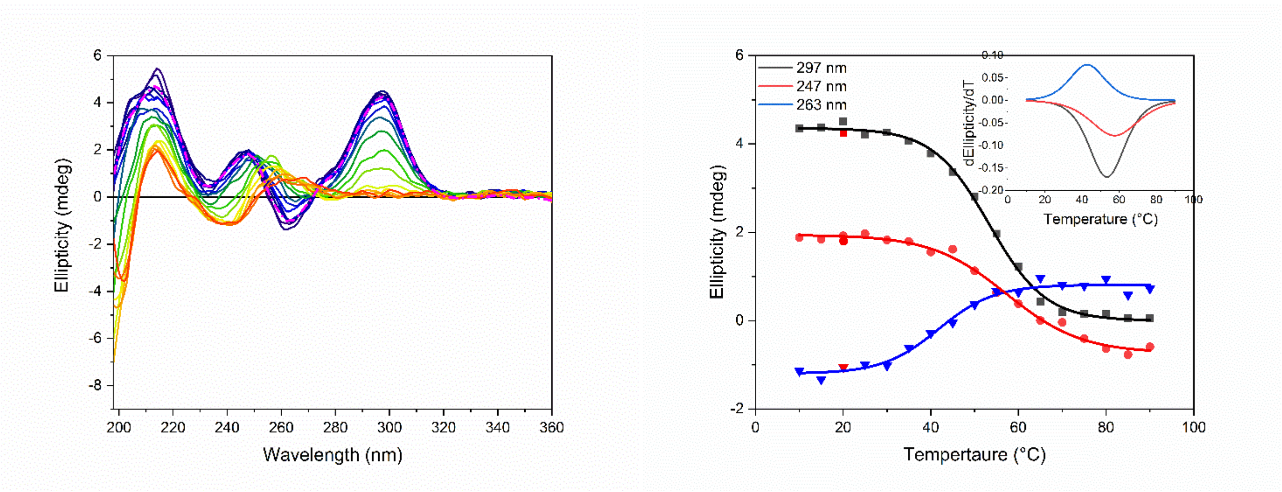

Figure S12. (**left**) Influence of temperature on the CD spectrum of *Htelo1* (13.5 μM) in 10 mM Tris-HCl buffer, pH 7.4, added of 70 mM NaCl. The temperature varied from 10°C to 90°C every 5°C. The dashed line represents the oligonucleotide cooled to 20°C after heating to 90°C. Spectra were acquired on module A of beamline B23 at Diamond Light Source syncrotron in a 0.1 cm pathlength quartz cuvette at 39 nm/min, DIT 1s, data pitch 0.5 nm, bandwidth 1 nm, in the 10-90°C range with steps of 5°C and allowing 2 minutes equilibration. (**right**) CD-melting curves at 247nm (red), 263nm (blue), and 297nm (black) with insert for comparison of the corresponding first derivatives.

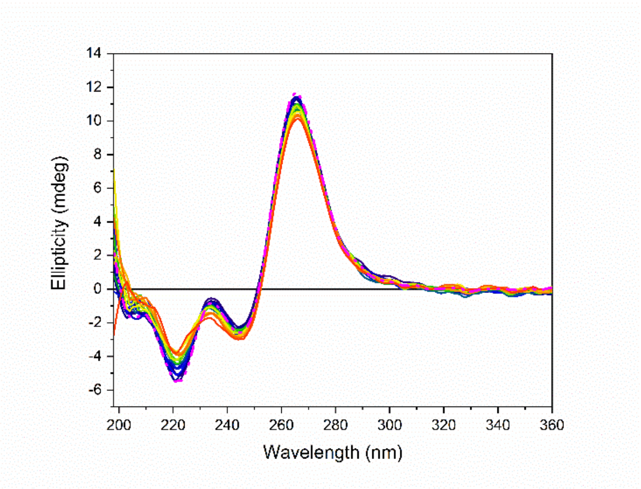

Figure S13. Influence of temperature on the CD spectrum of *T95-2T* (13.5 μM) in presence of 2.2 molar equivalents of ***Rhau25*** in 10 mM Tris-HCl buffer, pH 7.4, 70 mM KCl. Spectra were acquired on module A of beamline B23 at Diamond Light Source syncrotron in a 0.1 cm pathlength quartz cuvette at 39 nm/min, DIT 1s, data pitch 0.5 nm, bandwidth 1 nm, in the 10-90°C range with steps of 5°C and allowing 2 minutes equilibration.

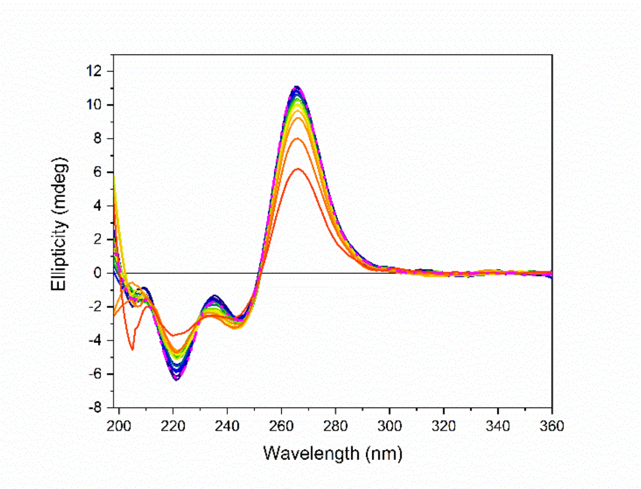

Figure S14. Influence of temperature on the CD spectrum of *T95-2T* (13.5 μM) in presence of 2.2 molar equivalents of ***Rhau25*** in 10 mM Tris-HCl buffer, pH 7.4, 70 mM NaCl. Spectra were acquired on module A of beamline B23 at Diamond Light Source syncrotron in a 0.1 cm pathlength quartz cuvette at 39 nm/min, DIT 1s, data pitch 0.5 nm, bandwidth 1 nm, in the 10-90°C range with steps of 5°C and allowing 2 minutes equilibration.

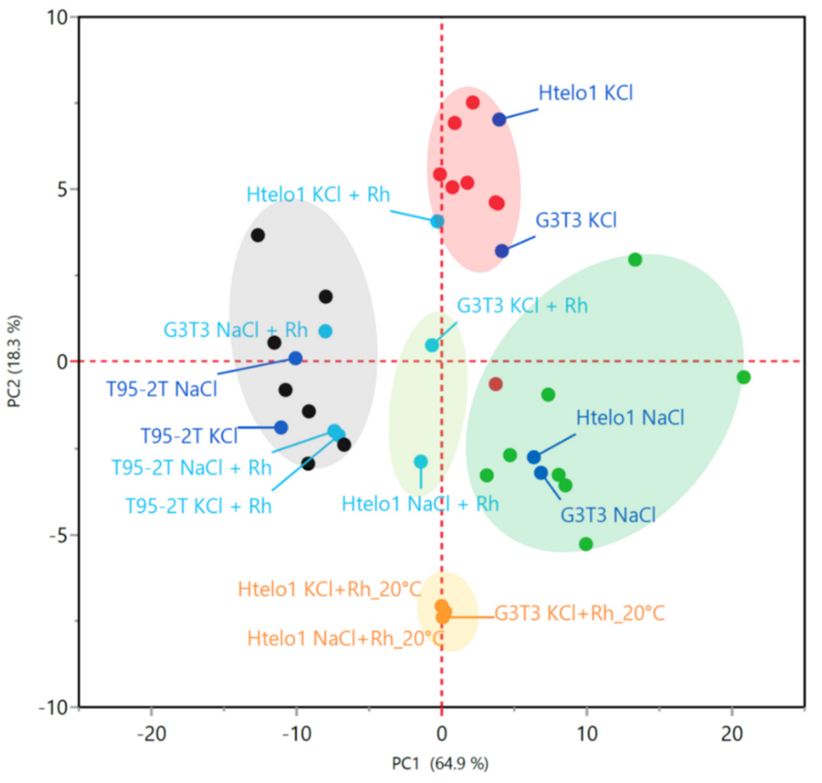

Figure S15. PCA plot (first and second principal components; in parentheses is the fraction of total variance explained) obtained with CD spectra of the experimental samples, analyzed together reference spectra from [Del Villar-Guerra *et al.*, *Angew Chem Int Ed Engl* **2018,** 57, (24), 7171-7175]. Blue points indicate the 12 samples of the present study; black points are reference data, amber points represent the data recorded at 20°C after the heating and cooling experiment for the samples that showed a conformational change. Grey, dark green, light and red ellipses show the groupings obtained according to [Del Villar-Guerra *et al.*, *Angew Chem Int Ed Engl* **2018,** 57, (24), 7171-7175], corresponding to parallel, antiparallel and hybrid G4 topologies, respectively. The analysis was carried out using the most distinctive interval of wavelengths (220-310 nm) of the spectra.

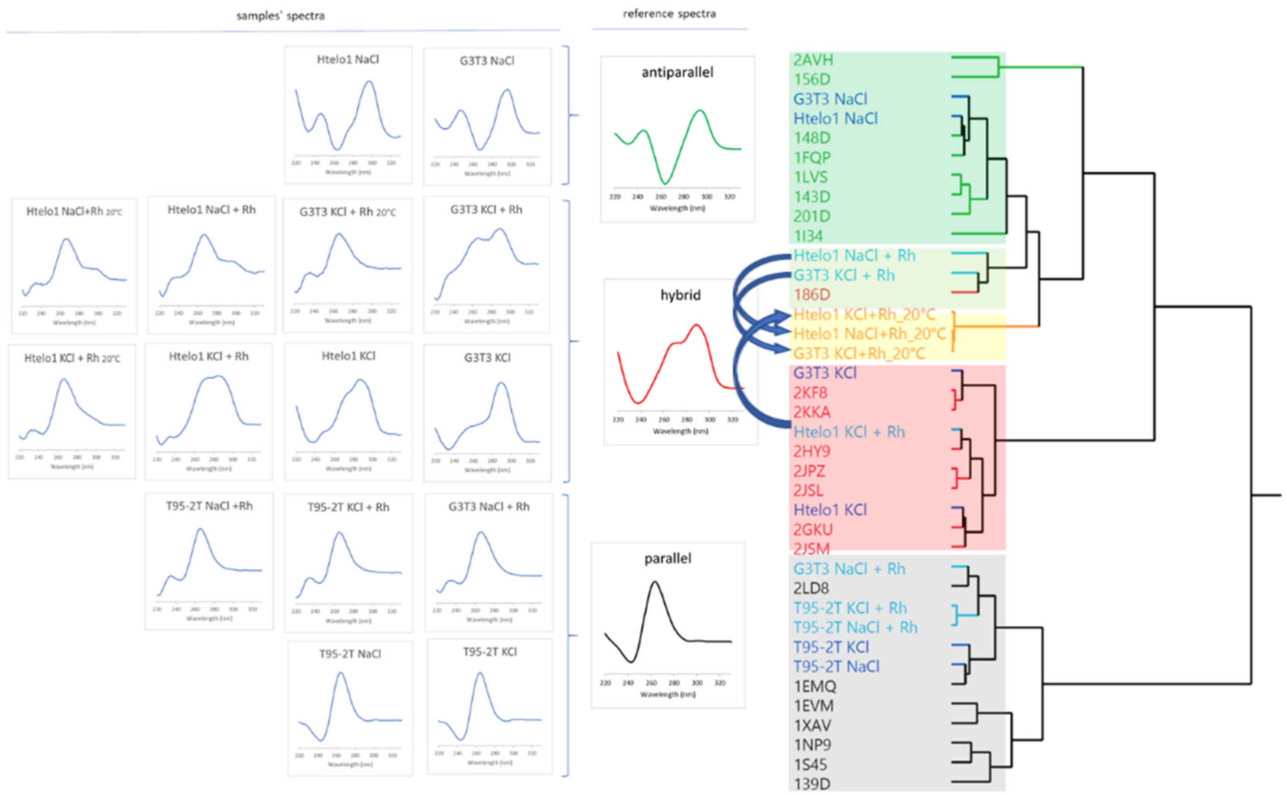

Figure S16. Hierarchical cluster analysis on the CD spectra of experimental samples, evaluated together reference spectra from [Del Villar-Guerra *et al.*, *Angew Chem Int Ed Engl* **2018,** 57, (24), 7171-7175]. The dendrogram on the right indicates three main clusters; for each cluster, a typical associated CD reference spectrum is shown, indicating parallel, antiparallel and "hybrid" G4 topologies, respectively. On the left, the spectra of all the experimental samples are reported, grouped according to cluster analysis. Data recorded at 20°C after the heating and cooling experiment for the samples that showed a conformational change allow to identify a further cluster (highlighted in amber color) which showed to have intermediate characteristics. Cluster analysis was obtained according to Ward's minimum variance method using the data of the most distinctive spectra interval (250-300 nm).

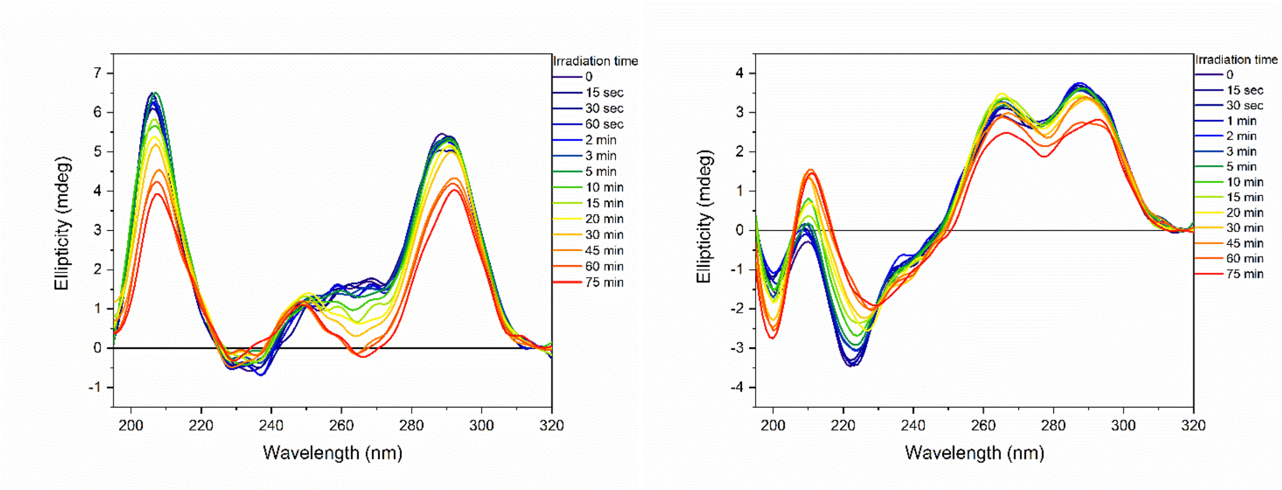

Figure S17. CD spectra of *G3T3* (13.5 µM) alone (**left**) or in presence of ***Rhau25*** (**right**) in 10 mM Tris-HCl and 70 mM KCl buffer. CD spectra were recorded at increasing UV irradiation times on a Jasco J1500 spectrometer, scan speed 100 nm/min, DIT 1s, datapitch 0.5 nm, bandwidth 2 nm, in a 1 mm pathlength quartz cuvette at 25°C.

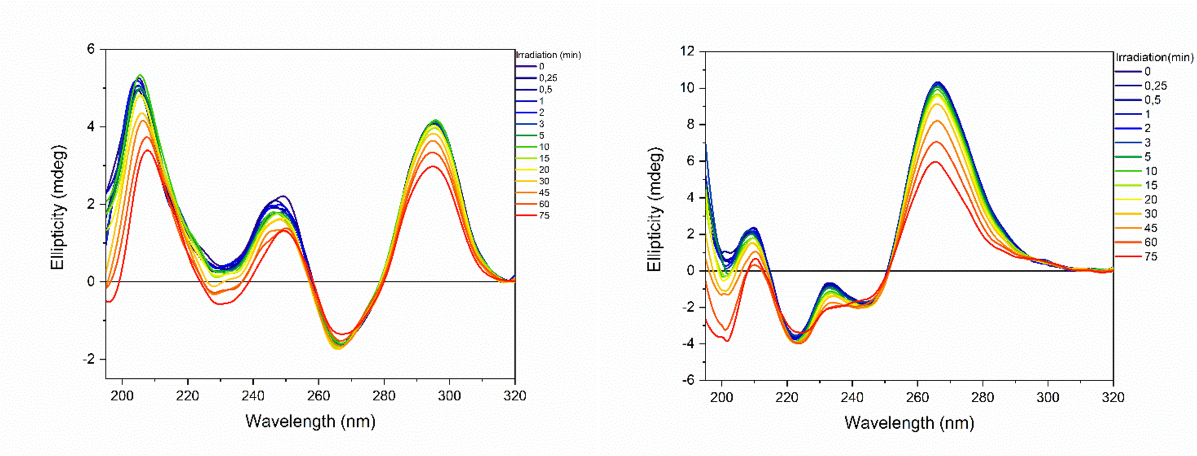

Figure S18. CD spectra of *G3T3* (13.5 µM) alone (**left**) or in presence of ***Rhau25*** (**right**) in 10 mM Tris-HCl and 70 mM NaCl buffer. CD spectra were recorded at increasing UV irradiation on a Jasco J1500 spectrometer, scan speed 100 nm/min, DIT 1s, datapitch 0.5 nm, bandwidth 2 nm, in a 1 mm pathlength quartz cuvette at 25°C.

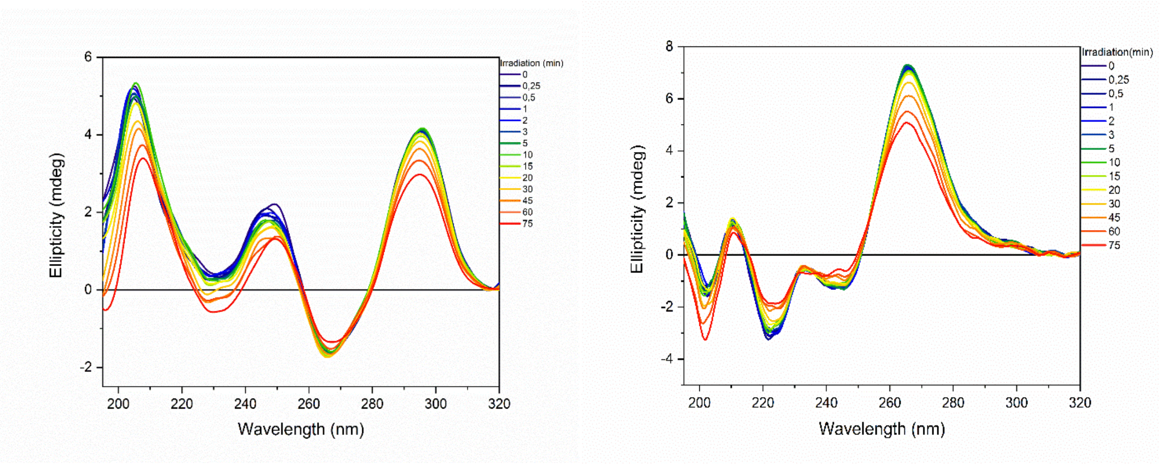

Figure S19. CD spectra of *Htelo1* (13.5 µM) alone (**left**) or in presence of ***Rhau25*** (**right**) in 10 mM Tris-HCl and 70 mM NaCl buffer. CD spectra were recorded at increasing UV irradiation times on a Jasco J1500 spectrometer, scan speed 100 nm/min, DIT 1s, datapitch 0.5 nm, bandwidth 2 nm, in a 1 mm pathlength quartz cuvette at 25°C.

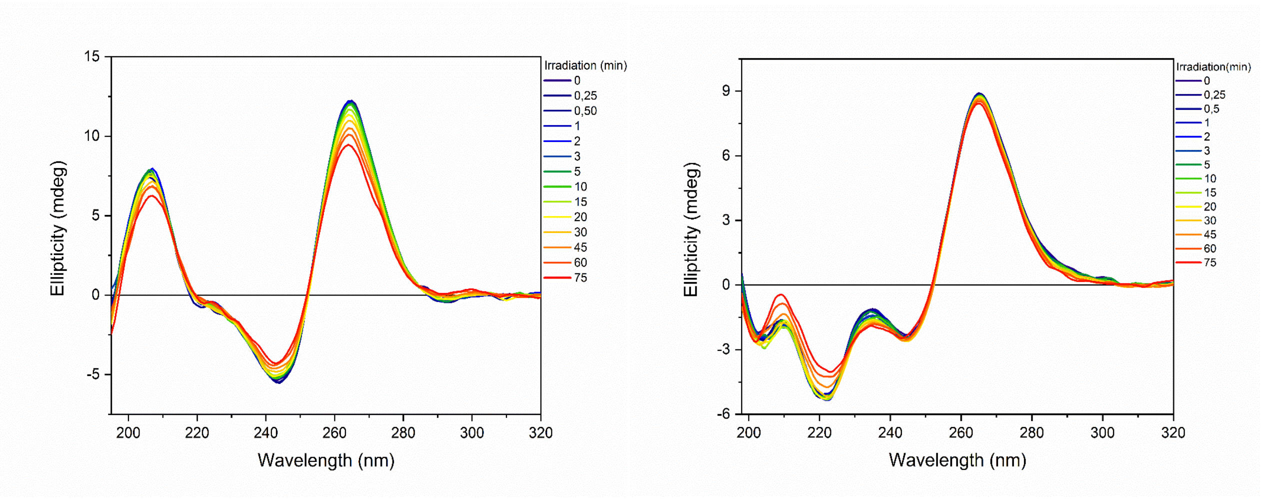

Figure S20. CD spectra of *T95-2T* (13.5 µM) alone (**left**) or in presence of ***Rhau25*** (**right**) in 10 mM Tris-HCl and 70 mM KCl buffer. CD spectra were recorded at increasing UV irradiation times on a Jasco J1500 spectrometer, scan speed 100 nm/min, DIT 1s, datapitch 0.5 nm, bandwidth 2 nm, in a 1 mm pathlength quartz cuvette at 25°C.

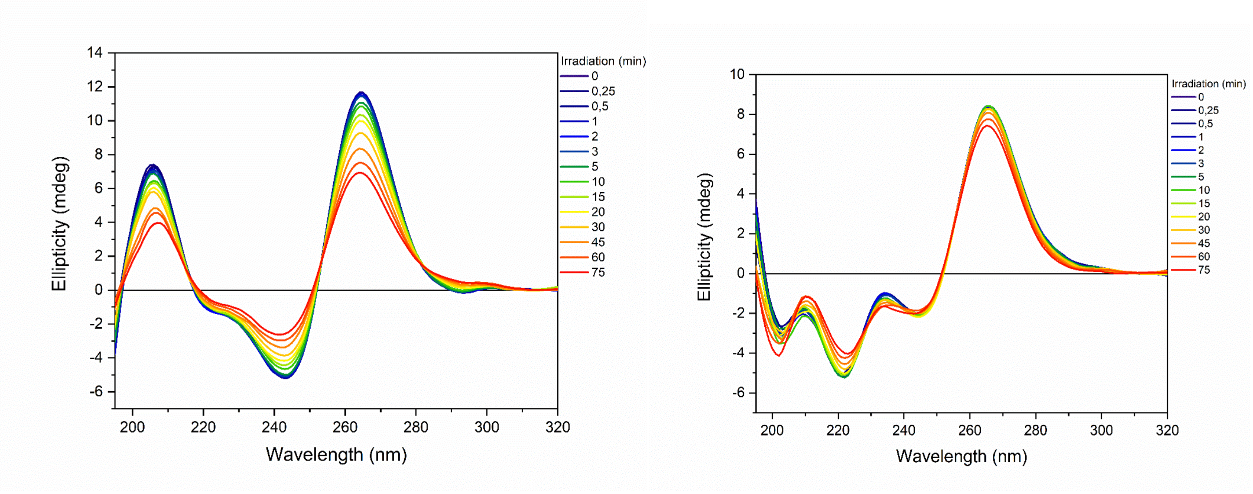

Figure S21. CD spectra of *T95-2T* (13.5 µM) alone (**left**) or in presence of ***Rhau25*** (**right**) in 10 mM Tris-HCl and 70 mM NaCl buffer. CD spectra were recorded at increasing UV irradiation times on a Jasco J1500 spectrometer, scan speed 100 nm/min, DIT 1s, datapitch 0.5 nm, bandwidth 2 nm, in a 1 mm pathlength quartz cuvette at 25°C.
